# Supporting young people through the COVID-19 pandemic and beyond: a multi-site qualitative longitudinal study

**DOI:** 10.1186/s12913-024-11752-z

**Published:** 2024-10-22

**Authors:** Madelyn Whyte, Emily Nichol, Lisa D. Hawke, Kelli Wuerth, Meaghen Quinlan-Davidson, Aileen O’Reilly, Joseph Duffy, Steve Mathias, JL Henderson, Skye Pamela Barbic

**Affiliations:** 1 Foundry, 1045 Howe Street, Vancouver, BC V6Z 2A9 Canada; 2 Centre for Addition and Mental Health, 1001 Queen Street, Toronto, ON M6J 1H4 Canada; 3 Department of Psychiatry, University of Toronto, 250 College Street, Toronto, ON M5T 1R8 Canada; 4 Jigsaw – The National Centre for Youth Mental Health, 16 Westland Square, Pearse Street, Dublin, D02 K535 Ireland; 5 Department of Psychology, University College Dublin, Belfield, Dublin 4, D04 V1W8 Ireland; 6 Providence Research, 10th Floor-1190 Hornby Street, Vancouver, BC V6Z 2K5 Canada; 7 Centre for Advancing Health Outcomes, 570-1081 Burrard Street, Vancouver, BC V6Z 1Y6 Canada; 8 Faculty of Medicine, Health Sciences Mall, University of British Columbia, Vancouver, BC 317-2194, V6T 1Z3 Canada; 9 Department of Psychiatry, University of British Columbia, 2255 Wesbrook Mall, Vancouver, BC V6T 2A1 Canada; 10 Department of Occupational Science and Occupational Therapy, T325-2211 Wesbrook Mall, University of British Columbia, V6T 2A1, Vancouver, T325-2211 BC Canada

**Keywords:** Youth, COVID-19 pandemic, Mental Health, Public Health

## Abstract

**Background:**

Throughout the COVID-19 pandemic, youth have experienced substantial stress due to abrupt changes in education, finances, and social life, compounding pre-existing stressors. With youth (ages 15–26) often at critical points in development, they are vulnerable to long-term mental health challenges brought on by pandemic trauma.

**Methods:**

To identify youth experiences throughout the pandemic and examine changes over time, we conducted semi-structured interviews among *n* = 141 youth in two Canadian provinces (Ontario and British Columbia) and across the country of Ireland at three time points over the course of more than one year (August 2020-October 2021). We conducted a qualitative longitudinal analysis using an inductive content approach.

**Results:**

Categories identified were (1) coping with hardship; (2) opportunities for growth; (3) adapting to new ways of accessing services; (4) mixed views on the pandemic: attitudes, behaviour, and perception of policy response; (5) navigating COVID-19 information; (6) transitioning to life after the pandemic; and (7) youth-led recommendations for government and service response. The findings also reveal trends in health and wellness in accordance with prolonged periods of lockdown, changes in weather, and return to normalcy after the availability of COVID-19 vaccines. Key recommendations from youth include incorporating youth voice into decision making, communicating public health information effectively to youth, enhancing service delivery post-pandemic, and planning for future pandemics.

**Conclusions:**

These results provide insights into the extensive longitudinal impacts of the COVID-19 pandemic on young people across three geographical locations. Actively involving youth in decision making roles for future pandemics or public health emergencies is critical.

**Supplementary Information:**

The online version contains supplementary material available at 10.1186/s12913-024-11752-z.

## Background

Researchers have long studied the relationship between risk and resilience to determine ways to promote positive health outcomes in trauma-exposed youth [1]. Literature exploring the impacts of terrorism and natural disasters has uncovered ripple effects for those exposed to traumatic events in history. Studies on youth mental health symptomology following the 9/11 terrorist attacks revealed links between direct exposure and heightened rates of post-traumatic stress disorder [2–4]. Similarly, data on youth affected by Hurricane Katrina found higher rates of depression and anxiety in the years following the disaster, with secondary stressors of loss of home and financial strain compounding mental turmoil [5, 6].

As with discrete traumatic events, the COVID-19 pandemic presented unique challenges for youth (defined here as ages 15–26 years) [7]. Youth reported disruptions in accessing social and recreational services and services for their mental and physical health [8]. For example, while mental health and substance use services quickly pivoted to virtual service delivery modalities, many end users reported not receiving needed services [8, 9]. Acute effects of the COVID-19 pandemic on youth include high rates of stress [10–12] and interpersonal challenges [13], gendered differences in mental health status [12, 14–17], and mixed effects on substance use [18–20]. Youth also experienced hardships with schooling and academic performance [21–24], with the transition to virtual learning posing challenges for many young people who had difficulties adjusting to a self-directed learning style and lack of supports [24]. Furthermore, the pandemic-related restrictions resulted in increased unemployment rates for youth ages 15–24 in all countries including Canada and Ireland, leading to financial strain and concerns about the future [8, 25]. Exposure to heightened stress through sudden risk of infection, school closures, financial strain, isolation, and an increasingly polarized political climate in the early stages of the pandemic created a tumultuous environment with the potential to affect lifetime developmental trajectories [10, 11, 26].

Quantitative longitudinal studies of youth mental health and wellbeing during the COVID-19 pandemic have suggested ongoing negative impacts of the pandemic as a whole and across many mental health and wellbeing variables [16, 17, 20, 27–29]. Stress, anxiety, and depression increased among youth during the pandemic, while general wellness and positive health behaviors such as physical activity declined [27, 28]. However, there is a lack of qualitative, longitudinal literature examining youth experiences during and perceptions of the COVID-19 pandemic and response. An in-depth exploration of youth experiences during the pandemic is required to gain insight into their mental health experiences, perspectives on public health measures, hopes and aspirations for the future, and recommendations for the post-pandemic recovery and future outbreaks, pandemics, and public health crises.

To gain insights into the ongoing experiences of youth during the pandemic internationally and understand how to best meet their needs going forward, we conducted a longitudinal qualitative study during the pandemic. Our objectives were to (1) explore youth experiences during COVID-19 at three time points and in three regions (two provinces in Canada: British Columbia and Ontario, and the country of Ireland) to determine ongoing impacts of the pandemic on mental health and wellbeing, and (2) offer youth-oriented recommendations for government and service response.

## Methods

### Design

This qualitative study aims to explore youth perceptions of the COVID-19 pandemic across three regions: British Columbia, Ontario, and Ireland and three time points. Employing a phenomenological approach, we sought to capture the lived experiences of young people and understand the meanings they ascribe to these experiences during this unprecedented time.

### Study sample

We recruited participants for this multi-phase longitudinal study from three geographic locations: Ireland and two Canadian provinces [British Columbia (BC) and Ontario (ON)]. Recruitment procedures varied slightly by site as described below. The selection of the three geographical locations was predicated on three key criteria: (1) each location implemented a comparable model of care tailored to youth; (2) established research infrastructure existed to facilitate a rapid response to the pandemic-related needs of youth; and (3) the sites shared analogous policies pertinent to pandemic management. We selected participants using purposive sampling to ensure representation across different demographics, including age, gender, and cultural backgrounds. Ethical approval for this study was provided by Jigsaw’s Research Ethics Committee (JREC/2020/004), the Centre for Addiction and Mental Health Research Ethics Board (046-2020), and the University of British Columbia Behavioural Ethics Research Board (H20-01537).

### Ireland

In Ireland, a mental health seeking sample was recruited from Jigsaw services. Jigsaw – the National Centre for Youth Mental Health is an early intervention integrated mental health service in Ireland that aims to support youth aged 12 to 25 with their mental health and wellbeing [30]. Clinicians invited youth ages 16–25 (with consent from parents/guardians, if under 18) who resided in Ireland and used Jigsaw’s brief intervention services to participate in the study, and, with consent, youths’ contact details were shared with a member of the research team. An invitation to take part in the study was also emailed to all youth registered for Jigsaw’s online synchronous chat support service who had provided consent to be contacted for research purposes. As per the protocol at Jigsaw, no honorarium was offered to participants recruited from the clinical sites. The rationale was that the payment could influence their help-seeking behaviours.

### Ontario (ON), Canada

In ON, youth ages 14–28 who were participating in a larger longitudinal quantitative study on mental health and COVID-19 at the Centre of Mental Health and Addictions (CAMH) [31] were asked for consent to be contacted for this sub-study. CAMH is the largest mental health teaching hospital in Canada, providing a range of clinical services for all ages. The study included youth recruited from three clinical studies and one non-clinical study [32–34]. Among those who provided consent, emails were sent to potential participants, and purposive sampling was carried out to include a diverse sample of youth in this sub-study. Thirty participants were recruited from the clinical sample, and thirty-one participants were recruited from the non-clinical sample, with the goal of representing both cohorts and a wide range of demographic characteristics. Participants received an email message inviting them to participate. Participants were paid a $30 honorarium for each interview.

### British Columbia (BC), Canada

In BC, a Foundry-led social media campaign was developed with youth and launched to recruit diverse youth ages 16–24 who resided in BC. Foundry is an integrated youth services initiative for youth ages 12–24 that provides support in person and virtually through a single access point for multiple service streams, including mental health [35]. Among those who indicated interest in the study, purposive sampling was carried out, and a diverse sample of youth from across the province was invited to the consent phase of the study. Two groups were recruited: A clinical group (*n* = 30) who had accessed Foundry services in the past 12 months, and a non-clinical group (*n* = 30) who had not accessed Foundry services. As with ON, participants in BC were paid a $30 honorarium for each interview.

### Consent and interview procedures

Data collection procedures at each site varied slightly. All procedures were conducted in English. Youth provided verbal or written informed assent/consent to their data being collected and stored for research purposes. In cases where youth could not legally consent due to jurisdictional laws, consent was received from their parent/guardian. Participants were informed that all questions were optional, and that they could end the interview at any time. Participants were followed up with via email. The interview schedule is reported in Table 1. Our research team comprises individuals both within and outside the gender binary.

**Table 1:** Interview schedule across all sites

	T1	T2	T3
Ireland	November 2020 – January 2021	February – March 2021	August 2021
Ontario	August 2020	March – April 2021	September – October 2021
British Columbia	November 2020 – January 2021	March – June 2021	August – October 2021

In ON, demographic data (e.g., age, gender identity, race/ethnicity, employment status) and psychosocial data were collected from the initial quantitative survey [31]. Interviews were conducted by phone or through a secure video conferencing system, WebEx (Cisco Systems, San Jose, California), hosted on a secure institutional server. Interviews were digitally recorded and transcribed verbatim by either a research staff member or a professional transcription agency. Participants were assigned unique participant IDs, and identifying information was removed during transcription. In Ireland and BC, demographic surveys were completed electronically at the start of all three time points. In BC, psychosocial questions were completed as part of this survey. Semi-structured interviews were conducted through Zoom (Zoom Video Corporation, San Jose, California) by a member of the research team. In BC, this research team for interviews consisted of a research coordinator and seven youth research assistants, and in Ireland the team consisted of two youth research assistants. Youth research assistants were between the ages of 16–24 years, who received training and supervision from the study leads (SB, JH/LH, AO) to conduct interviews and support analyses. Interviews were recorded and transcribed verbatim by two youth research assistants in BC and two youth research assistants in Ireland. Unique participant IDs were given and identifying information was removed from the transcripts to maintain confidentiality. A copy of the semi-structured interview guides used across all sites is contained in Appendix A.

The engagement of youth was a core component of this study. One to two youth co-researchers were employed at each site to support all elements of the research process and were provided with training and supervision by the site lead researchers. Youth co-researchers were aged 18–24 years, had lived and living experience of mental health challenges and accessing the system, had past research experience in health services research about youth, and were hired by the local site leads. Youth co-researcher groups were consulted on various aspects of the project across the three study sites, including co-developing the recruitment materials and research and interview questions, interpreting and validating the findings, and contributing to other knowledge translation materials [36, 37].

### Analysis

We employed inductive content analysis, which involved reading and re-reading the data (familiarisation); identifying broad categories; developing and refining subcategories and fine-grained codes; and collating and interpreting the data to generate succinct categories and subcategories [38]. We used NVivo 12 Software [39] to support data analysis. This process was iterative, with many drafts of refinements until an accurate depiction of the data was captured [38]. At the intra-site level, the research team met weekly to ensure a mutual understanding of code definitions and coded two transcripts together for each time point. At these meetings, study site leads (MW/SB, AO, LH) reviewed the codes and resolved conflicts if they occurred. At the inter-site level, all team members met weekly to discuss discrepancies and refine codes and the coding framework. For this paper, we present site differences over time. A future analysis is planned to explore differences in age, gender, and race/ethnicity amongst the categories and will be reported elsewhere.

## Results

### Demographics

As shown in Table 2, a total of 141 participants ages 14–26 years took part in the study at T1 (BC *n* = 59, ON *n* = 61, Ireland *n* = 21). Retention was 87% at T2 (*n* = 123 total; BC *n* = 50, ON *n* = 56, Ireland *n* = 17), and 78% at T3 (*n* = 110 total; BC *n* = 43, ON *n* = 53, Ireland *n* = 14). The interview times ranged from 20 to 120 min. On average, participants from Ireland were younger, with 33.3% of youth aged 14–17, compared to BC (20.3%) and Ontario (6.6%). Participants from Ontario had more gender diversity (26.1% boys/men, 47.5% girls/women, 16.3% outside the gender binary) compared to BC (72.9% girls/women) and Ireland (85.7% girls/women).

**Table 2:** Sociodemographic characteristics of participants included in the study at T1

	BC (*n* = 59)	ON (*n* = 61)	Ireland (*n* = 21)	Total (*n* = 141)
Age (years)				
14–17	12 (20.3%)	4 (6.6%)	7 (33.3%)	23 (16.3%)
18–21	27 (45.8%)	30 (49.2%)	10 (47.6%)	67 (47.5%)
22–25	20 (33.9%)	26 (42.6%)	4 (19.1%)	50 (35.5%)
26	0 (0%)	1 (1.6%)	0 (0%)	1 (0.7%)
**Gender**				
Boys/Men	12 (20.3%)	22 (36.1%)	2 (9.5%)	36 (25.5%)
Girls/Women	43 (72.9%)	29 (47.5%)	18 (85.7%)	90 (63.8%)
Non-binary	0 (0%)	6 (9.8%)	1 (4.8%)	7 (5.0%)
Transgender	1 (1.7%)	1 (1.6%)	0 (0%)	2 (1.4%)
Another gender identity	3 (5.1%)	3 (4.9%)	0 (0%)	6 (4.3%)
**Race/Ethnicity**				
Caucasian	25 (42.4%)	24 (39.3%)	17 (81.0%)	66 (46.8%)
Black	0 (0%)	6 (9.8%)	2 (9.5%)	8 (5.7%)
Asian	18 (30.5%)	20 (32.8%)	2 (9.5%)	40 (28.4%)
Latin American	1 (1.70%)	1 (1.6%)	0 (0.0%)	2 (1.4%)
Mixed heritage	8 (13.6%)	8 (13.1%)	0 (0.0%)	16 (11.4%)
Ashkenazi Jewish	0 (0.0%)	1 (1.6%)	0 (0.0%)	1 (0.7%)
Middle Eastern/North African	5 (8.5%)	0 (0.0%)	0 (0.0%)	5 (3.6%)
Métis	1 (1.7%)	0 (0.0%)	0 (0.0%)	1 (0.7%)
Missing	1 (1.7%)	1 (1.7%)	0 (0.0%)	2 (1.4%)

Table 3 outlines psychosocial factors documented across time-points in BC and ON. Due to resource limitations, participants from Ireland did not complete the psychosocial surveys.

**Table 3:** Psychosocial characteristics across time points in BC and ON*

	BC			ON		
Employment Status	**T1 (** *n* ** = 59)**	**T2 (** *n* ** = 41)**	**T3 (** *n* ** = 27)**	**T1 (** *n* ** = 60)**	**T2 (** *n* ** = 54)**	**T3 (** *n* ** = 51)**
Employed	10 (17.0%)	9 (22.0%)	7 (25.9%)	6 (10.0%)	14 (25.9%)	16 (31.4%)
In school	24 (40.7%)	14 (34.1%)	4 (14.8%)	35 (58.3%)	16 (29.6%)	12 (23.5%)
Both	23 (39.0%)	16 (39.0%)	12 (44.4%)	9 (15.0%)	19 (45.2%)	20 (39.2%)
Neither	2 (3.4%)	2 (4.9%)	4 (14.8%)	10 (16.7%)	5 (9.3%)	3 (5.9%)
Physical Health	**T1 (** *n* ** = 59)**	**T2 (** *n* ** = 41)**	**T3 (** *n* ** = 27)**	**T1 (** *n* ** = 59)**	**T2 (** *n* ** = 54)**	**T3 (** *n* ** = 51)**
Poor to Fair	9 (15.3%)	8 (19.5%)	3 (11.1%)	19 (32.2%)	20 (37.0%)	18 (35.3%)
Good to Excellent	50 (84.8%)	33 (80.5%)	24 (88.9%)	40 (67.8%)	34 (62.7%)	33 (64.7%)
Mental/Emotional Health	**T1 (** *n* ** = 59)**	**T2 (** *n* ** = 41)**	**T3 (** *n* ** = 27)**	**T1 (** *n* ** = 59)**	**T2 (** *n* ** = 55)**	**T3 (** *n* ** = 51)**
Poor to Fair	33 (55.9%)	19 (46.3%)	10 (37.0%)	28 (47.5%)	23 (41.8%)	22 (43.1%)
Good to Excellent	26 (44.1%)	22 (53.7%)	17 (63.0%)	31 (52.5%)	32 (58.2%)	29 (56.9%)

* Some participants did not fill in demographics at all time points and some surveys were partially complete

## Content categories

Table 4 outlines seven primary categories and multiple subcategories uncovered during analysis. Interviews at different time points and sites were analyzed separately and trends over time and across sites were documented, capturing shifts in health and wellness, personal circumstance, and perception of policy response. These findings are discussed together when consistent across sites and time points, and differences are described when applicable. These categories and subcategories are described in the following sections and include verbatim supporting quotes

**Table 4:** Categories and subcategories

**Categories**	**Subcategories**
1. Coping with hardship	1.1 Experiences of loss and missed opportunities 1.2 Isolation, cohabitation, and interpersonal challenges 1.3 Disruption to education and professional development 1.4 Employment and financial setbacks 1.5 Mental and physical health decline 1.6 Urban vs. rural living 1.7 Experiences of xenophobia
2. Opportunities for growth	2.1 Learning new skills and hobbies 2.2 Slower pace of life and commitment to personal development 2.3 Gratitude for re-opening and life prior to pandemic 2.4 Positive experiences with remote education and employment
3. Adapting to new ways of accessing services	3.1 Difficulties seeking support in the midst of health service disruptions 3.2 Getting comfortable with virtual services
4. Mixed views on the pandemic: Attitudes, behaviour, and perception of policy response	4.1 Adherence to guidelines 4.2 Perception of policy response 4.3 Absent youth voice in policymaking 4.4 Unfair judgement of youth role in the pandemic 4.5 Vaccination in the face of hesitation
5. Navigating COVID-19 information	5.1 Confusion around pandemic information presented by the government 5.2 Accessing COVID-19 information through multiple forms of media
6. Transitioning to life after the pandemic 7. Youth-led recommendations for government and service response	6.1 Cautious readjustment to a new normal 6.2 Concerns for the future 6.3 Optimism about positive social changes 7.1 Youth voice needed in decision making 7.2 Communicating public health information effectively to youth and combatting misinformation 7.3 Post-pandemic services: enhancing service delivery to improve outcomes 7.4 Planning for future pandemics

### Category 1. Coping with hardship

The study revealed a range of pandemic-related hardships that contributed to significant cumulative stress over the course of the investigation. Participants articulated a multitude of challenges, including the loss of cherished experiences, strained interpersonal relationships, disruptions to education and professional development, financial setbacks, deteriorating mental health, and increased xenophobia fueled by rising political tensions. These interconnected issues are described in seven sub-categories.

#### Experiences of loss and missed opportunities

Youth reported experiencing loss of important childhood and adolescent milestones, describing missing out on their *“prime years”* (BC, T1) due to lockdowns and restrictions. Youth expressed awareness for how their generation has been impacted in unique ways due to the transitional nature of the participants’ ages, with one participant saying, *“I’ve been a lot more lonely … it feels like a ton of my prime-time – my prime years are being wasted”* (BC, T1). For some, these disruptions felt like a disadvantage in terms of emotional and professional development, with one participant saying, *“It affects our generation a lot*,* just because … these are our prime years to meet new people and gain new experiences and stuff like that. I feel like we have … a disadvantage”* (BC, T1).

Youth were also impacted by missing out on important events to celebrate achievements such as graduation, birthdays, and other moments of significance. These missed opportunities to recognize pivotal moments of accomplishment in a public forum were realized in the early days of the pandemic: *“I didn’t have a graduation ceremony*,* so I didn’t get to celebrate or take pictures with my parents. Didn’t get to walk with my diploma or say goodbye to my friends*,* or do any form of partying or celebrating finishing school”* (BC, T1).

No longer able to engage in typical activities as an adolescent, youth suffered erasure of formative experiences, reporting, *“you’re never going to get the year back”* (Ireland, T2). Lost time and missed opportunities contributed to a collective sense of grief.

#### Isolation, cohabitation, and interpersonal challenges

Across regions, interpersonal challenges became apparent due to lockdown and restrictions on social gatherings, where connections became strained due to diminished contact: *“It is almost like a whole year of bonding is gone”* (Ireland, T1). Warmer weather acted as a buffer against isolation, since participants had more freedom to engage with others safely outside. During colder months, as case numbers rose and restrictions tightened, participants expressed increased feelings of loneliness:

Different views on policy guidelines and pandemic restrictions contributed to relational disconnect. The strain on relationships was most apparent at T2 as restrictions tightened, and friends and family members had differing views on vaccinations and following guidelines:

“*I still feel kind of disconnected from a lot of my friends*,* just because of the pandemic*,* and everyone’s different ways that they feel about the restrictions. A lot of my friends are*,* maybe*,* more open*,* and maybe not following the rules as much as myself”* (BC, T2).

Many participants expressed technology fatigue over time, where communicating in the form of texting or video chatting could not replace the in-person connection: *“That’s a big part of it*,* not being able to just hug people”* (Ireland, T2). For some, maintaining virtual relationships was a daunting task, described more like a chore than an opportunity for social interaction: “*You kind of can only keep so many relationships going virtually”* (ON, T2). As one participant described the trials and tribulations of virtual communication:

*“I just have a lot of troubles with calls*,* maybe not calls*,* but texting people … I just don’t think you can maintain any sort of social relationship just on social media.*” (ON, T1).

In contrast, too much time with family was also reported by some participants to fuel conflict due to a lack of personal space, with youth noting the adverse effects on their mental health as a result. *“Family issues spiked up”* (Ireland, T1) during lockdown, when family members were living, working, and attending school under one roof, the effects of which were felt most among youth with challenging dynamics with their parents: *“My parents weren’t very supportive of me and I kind of had to stay in that house*,* and you know*,* get talked down to and belittled every single day*,* but also my anxiety from not going out really anywhere skyrocketed”* (ON, T1). Confined by the inability to seek refuge in safe relationships, feelings of isolation were compounded for youth in challenging family dynamics.

#### Disruption to education and professional development

Youth described how online learning was an adjustment. Difficulties associated with abrupt changes in educational formats were most apparent at T1. Youth experienced obstacles managing a self-directed learning style, feeling distracted and less productive at home. Some students with disabilities experienced challenges navigating a new way of learning without the necessary infrastructure to provide accommodations in virtual course formats:

*“A lot of institutions took the opportunity of digital and online services to mean that they no longer had to provide specific disability services*,* which*,* had they talked to students*,* would know that’s not acceptable nor equitable access … online does not mean accessible”* (BC, T1).

Technical issues compounded by adjusting to an online format while at home posed several learning barriers:

*“I find it very hard it’s like school online takes a huge toll on like me personally I find it extremely stressful to keep up with it. It’s given me a couple of like breakdowns especially in the last couple of weeks … it doesn’t feel like you’re actually in school like or in college. You’re just in a room watching things”* (Ireland, T1).

For those experiencing greater mental health problems due to pandemic-related challenges, academic performance declined. Students described heightened stress, lower mood, lack of focus, an inability to complete course work, and an overall work environment not conducive to productivity. Teacher-student communication issues emerged, where students needing extra assistance were unable to receive proper support virtually. Managing questions over email was not adequate for those requiring clarity and a fluid back-and-forth dialogue on an academic issue:

*“We used to speak with the teacher*,* we used to be in a classroom*,* you could talk to them. But now everything’s online*,* and learning online is a lot harder*,* ‘cause you can’t really ask questions the way you could in class sitting in front of the teacher”* (ON, T1).

In applied programs and career paths requiring more technical training among college- and university-aged students, educational progress slowed. Without adequate hands-on experience, youth felt they were not receiving the same type of education they would have had if the pandemic had not happened. In some cases, youth worried how lacking such skills would affect future career prospects, feeling *“ripped off in terms of professional development”* (BC, T2).

*“Starting a brand-new program*,* especially one that’s super hands-on learning*,* it was really difficult to find times and opportunities to practice hands-on skills that I need to develop”* (BC, T2).

#### Employment and financial setbacks

Job loss or reduced *“COVID hours”* (BC, T2) led to financial stress that was particularly noted in T1. Fear surrounding being able to find a new job and afford the cost of living took a toll emotionally, adding new stress for some on relationships:

*“It was the end of my school term*,* so I had no money left*,* and it was definitely pretty scary*,* so I don’t want to be in that situation where I was floundering*,* looking for money*,* and I ended up having to rely on my boyfriend a lot. Which*,* this is my ex-boyfriend*,* but it kind of created this weird power imbalance in our relationship*,* where I had to rely on him for everything”* (BC, T1).

As an age group that occupies a large portion of customer service and hospitality positions, youth expressed concern about returning to a work environment with potentially increased exposure to COVID-19: *“[Working in retail is] absolutely terrifying*,* to be exposed to that many people every day*,* like*,* you know*,* how many people come through to shop which is something that we’ve all been avoiding over the past couple months”* (Ireland, T1). Fear of contracting COVID-19 arose, along with challenges dealing with the public with the implementation of new public health mandates requiring masks and vaccination in certain establishments:

*“Since everything was opening up*,* the public seemed a little more lax in terms of some of the public health restrictions. So*,* you know getting into disagreements over*,* you know if they should wear a mask and stuff like that*,* and having to explain that the pandemic is still going on. It is a customer service job*,* so I’d get yelled at quite a lot this summer [2021] over that*,* as well as you know trying to enforce public health recommendations”* (ON, T3).

Mental health challenges presented in the form of disrupted sleep schedules, increased substance use and social anxiety, and decreased motivation. Youth described how prior stressors were exacerbated by pandemic-related stress, with symptoms most prevalent during lockdown periods and winter months.

With online school and reduced work hours or job loss, maintaining a routine became difficult, which perpetuated mental health challenges: *“I have trouble with maintaining a regular sleep schedule. And so that’s exacerbated by not having a schedule – like during the pandemic there’s more onus on me to schedule things and there’s less outside – outside factors for me to rely on as a schedule”* (ON, T2). Youth described reliance on unhealthy coping mechanisms and increased substance use to mitigate mental health challenges: *“When I feel like that [out of control]*,* then that’s when I start to smoke and do a lot of other things to keep myself distracted and mellow it out”* (ON, T1). Unable to turn to typical coping strategies such as leaning on friends, youth struggled to find ways to support their wellbeing:

*“My substance use has definitely gone up or it has become more frequent*,* like quite dramatically. And that’s in part because I*,* again*,* I live alone*,* I work from home*,* so there’s a lot more opportunity sort of for me to engage with substance use.* (ON, T2).

Over time, youth described how isolation heightened social anxiety: *“You’re always on edge*,* everything seems like we’re in war or something and you have to stay inside ‘cause there’s curfews and danger and everything all the time”* (Ireland, T2). Living in a perpetual state of fear when interacting with others and experiencing lockdowns led to difficulties socializing: *“Being cooped up inside 24/7 isn’t good for anybody … You stay inside*,* you’re going to develop different problems socially”* (Ireland, T2). Everyday tasks such as going to the grocery store or seeing friends became associated with fear:

*“I’m afraid of going into public places like Costco*,* or being in a bigger group of friends. If there’s ten people – even if we’re social distancing*,* I still feel uncomfortable if someone’s gone somewhere*,* and I know where they’ve gone*,* or I don’t know where they’ve gone even*,* I feel uncomfortable”* (BC, T2).

Motivation to learn, work, or complete typical tasks decreased while navigating life through the mundaneness of the pandemic: *“I can’t bring myself to do anything … If I tried to do school*,* I couldn’t do school*,* even one course. I can barely take care of myself throughout the day. I just don’t have any motivation to do anything”* (ON, T3).

For many, winter months intensified boredom, where shorter days and colder weather negatively impacted mental health. Youth described feeling like they were living the same day on repeat:

*“It definitely got worse in the winter. …I kind of started to feel like I was living the same day over and over again*,* especially in*,* like January*,* February*,* like I was living the same week because it would be like I have the exact same worries on Monday and then that’s done. And then I have the exact same worries again and again and again and again”* (Ireland, T2).

In instances where youth contextualized prior difficulties with mental health problems, COVID-19 added *“this extra layer of anxiety and stress that’s just been coated across everything … It feels like it’s just blanketed the whole thing*,* and it’s added more weight to everything”* (BC, T1). Youth described feeling a *“constant high state of alert and stress”* (BC, T2), with little to no relief: *“It’s just something that’s always there and I feel like it kind of accentuates whenever something that you do have that’s more negative on your life … It kind of compounds with anything negative in your life*,* and so it’s very easy to kind of wear down on you”* (ON, T2).

In addition to negative mental health impacts of the pandemic, youth experienced physical health declines due to closure of gyms and recreation centres, colder weather making it difficult to exercise outdoors, and inaccessible physical health services, including doctors, physiotherapy, and other health services: *“I loved going to the gym but I have not been to a gym since probably December”* (ON, T2).

However, improved mental and physical wellbeing were often observed in summer months, where youth had opportunities to get outside, exercise, and safely interact with peers in a physically distanced manner: *“I think summer is also a big factor*,* like weather. It changes everyone’s mood. So I think it’s definitely more hopeful when the summer comes. Everyone will be more happier and more hopeful”* (BC, T2).

#### Youth living in urban vs. rural settings

COVID-19 experiences differed for youth depending on their community. Those in rural areas expressed gratitude for more open space to get outside and physically distance:

*“If I was to live in town in the city*,* I wouldn’t be going out for a walk*,* because I’d be more likely to bump into people … I’d be inside more*,* and then I’d be driven more crazy. I really do need those walks every now and again*,* and just get out of the house. And I don’t know if I would be confident enough to do that in town”* (Ireland, T2).

However, misinformation and people not following guidelines was a reported concern, with one youth in a rural community reporting, *“I think in terms of the general attitude around COVID*,* it’s maybe a bit more … Uneducated*,* than in a city”* (BC, T2). In urban spaces, greater population density led to an increase in fear and added challenges getting around the city:

*“If we had enough money to afford a car*,* we wouldn’t have to worry about catching COVID from public transit. Wouldn’t have to get in that sardine bus. We’d be able to go and visit friends without worrying about*,* like*,* OK*,* I am exposing myself to*,* like*,* hundreds of people”* (ON, T3).

#### Experiences of xenophobia

Asian-Canadians experienced a *“big increase in harassment and racism”* (BC, T2) noted midway through the study period. Instances of hateful rhetoric became more commonplace, and as a BC participant recalled, *“I did get some of the racist comments when I was at Costco*,* because of being Asian. I did receive a lot of comments about it*,* so I think those did affect me*,* and I think it’s going to affect me after the pandemic*,* as well”* (BC, T2). Because of these experiences, as well as seeing cases of racism and xenophobia on the news, some described feeling unsafe in public settings:

*“I guess it’s important to stay more vigilant now. (…) [S]ometimes there’s those kind of creepy looking [people] who you don’t know if they’re going to start verbally harassing you on the subway and stuff because you’re alone and there’s not people around to kind of protect you anymore”* (ON, T3).

In Ireland, one participant spoke about how xenophobia is resulting in a “*divide in society*”, citing a mentality in Irish society of *“‘No Chinese*,* no Brazilians*,* no Brits’*,* because like they brought the new variants”* (Ireland, T2). They also spoke about their family members experiences’ *“getting abused on the bus”* and being told “*you brought COVID here*” (Ireland, T2).

### Category 2. Opportunities for growth

This category emerged consistently among youth across all three recruitment locations. At Times 1 and 2 (T1 and T2), participants expressed a strong appreciation for the opportunity to acquire new skills and engage in a more measured approach to goal-setting and personal development. As the study progressed, youth participants articulated a growing sense of gratitude for previously taken-for-granted resources (T3), such as the re-opening of schools and recreational centers. Additionally, they acknowledged the adaptive measures their networks implemented during the pandemic, notably highlighting the efforts of teachers in developing innovative online curricula.

#### Learning new skills and hobbies

The pandemic presented an opportunity to develop new hobbies and skills. Honing a craft, indulging in creative endeavors, and exercising buffered against stressors in the early days of quarantine during T1 interviews:

*“During the first lockdown*,* I actually picked up the hobby of running*,* because I didn’t have anything else to do. And I always wanted to try running*,* but I never really got the chance to”* (Ireland, T1).

With newfound leisure time, youth were able to put their energy towards activities that would otherwise not be prioritized, which improved mental health:

*“Especially during the early days of quarantine*,* that was pretty helpful*,* being able to create something*,* or lots of people got into baking. I don’t know*,* I think little things like that*,* like being able to acquire a new skill that you didn’t have before*,* I think that’s important for your mental health*,* and just feeling like you are accomplishing something*,* even when everything else is kind of at a standstill”* (BC, T1).

#### Slower pace of life and commitment to personal development

Many used their newfound free time to slow down, self-reflect, and *“build better habits”* (ON, T3). The impacts of a slower pace of life were particularly apparent during T1 interviews, as some youth experienced reduced responsibilities because of changes in employment and education. This gave youth time to reflect: *“It’s forced me to slow down*,* in a way*,* and to spend more time with myself*,* and learn more about myself”* (BC, T1). Others were able to recover from burnout: *“I was running at such a high level*,* and burning a lot of steam and not giving myself that time to reflect on situations and myself*,* and COVID kind of forced me to do that*,* because I had the literal time to do it”* (BC, T1).

Despite hardship during the pandemic, more time alone acted as a catalyst for change: *“It taught me a lot about my mental health … how to take care of myself*,* and how to practice self-care”* (BC, T1). Several youth took initiative to understand themselves better and begin a journey of healing during this time.

In some instances, youth noted an increase in help-seeking behaviour, declaring, *“If it wasn’t for the pandemic*,* I think I’d still probably be suffering from mental health [problems]”* (Ireland, T3). These participants practiced self-care by confronting their mental health issues and reaching out for support instead of suppressing emotions.

*“The thing about being stuck in your house is that you can’t escape anything. Like all of your issues*,* you have to deal with them right now because there’s no going anywhere… I’m at a point now where I’m being forced to make life changes and I’m being forced to actively confront my life and my issues”* (Ireland, T2).

#### Gratitude for re-opening and life prior to the pandemic

Gratitude and appreciation toward returning to a“normal” life as restrictions lifted were noted across all time points:* "I think it kind of just made me appreciate everything, kind of more. Like even whenever I was able to see my granny for the first time, I just would be something so normal to me and then it was just like this whole big thing”* (Ireland, T1). For some, the pandemic served as a reminder to *“[have] gratitude for what’s happening in the present cause you don’t really know what’s going to happen in the future”* (ON, T3).

#### Positive experiences with remote education and employment

While some youth experienced a multitude of difficulties adjusting to remote education and employment, others found they were able to excel with the shift to virtual. Positive attitudes towards remote education and employment increased in T2 and T3 as rocky transitions passed. Virtual education and employment were valued for accessibility reasons for individuals who live in rural regions and/or have certain disabilities or physical or mental health conditions that limit their ability to participate in in-person activities: *“I also have a chronic illness*,* which affects fatigue and mobility*,* so being able to have class online was really helpful*,* as well”* (BC, T1).

### Category 3. Adapting to new ways of accessing services

Physical health, mental health, and substance use services had to quickly shift from in-person to virtual care. During this transition period, youth experienced service disruptions, noted in T1 interviews. Barriers to accessing services improved with time as adaptations to online service delivery developed. However, concerns including prolonged wait times, not feeling prioritized in the mental health sector, and hesitancy to seek help persisted through all time points.

### Difficulties seeking support in the midst of health service disruptions

 For many, wait times were an obstacle prior to the COVID-19 pandemic, made worse by service closures during lockdowns. Such disruptions left youth with few resources to manage their mental health: *“There was no options. There was no Skype call offered to me. There was literally nothing. I was lucky to receive a text message at one stage”* (Ireland, T2).

With nowhere to turn, several youth noticed a decline in health:

*“For myself*,* though … Maybe some worsened health*,* just because appointments have taken so long to get to*,* and I haven’t been able to see in-person doctors”* (BC, T1).

#### Getting comfortable with virtual services

Once virtual services were implemented, some youth expressed preference for in-person support, though many acknowledged benefits of virtual appointments. One was using virtual services for general health concerns and prescription refills:

*“It’s been working well*,* for me. Most of the time*,* it’s just for a prescription refill or an update on how my health has been*,* so it’s pretty convenient to just call and expect a phone call*,* like I can be at home*,* and I don’t have to go anywhere and wait for too long”* (BC, T1).

Another benefit was that many participants felt more comfortable accessing virtual supports than in-person supports due to the online disinhibition effect:

*“When I was doing it face to face I would shy away from a lot of conversations because I’d feel embarrassed or I’d get distracted. Where now on a Zoom call it just kind of feels like a normal phone call.”* (Ireland, T1).

By T3, the convenience and accessibility aspects of virtual services were acknowledged as something that should be continued: *“I think online services can be great*,* more accessibility for people in remote locations. Jobs that don’t conform to like business hours for therapists … I think it’s better than only in person*,* because in person is just not accessible”* (BC, T3).

On the other hand, some participants had ongoing negative experiences with virtual services. The effects of their day-to-day life being virtual began to take a toll: *“I’m so fatigued from all the virtual stuff that it would just drive me crazy if I tried to use virtual services for mental health”* (ON, T3).

For some participants in both countries, technology problems created fractures in the flow of appointments, negatively impacting the overall quality and experience of services:

*“When the network is cut off or the internet’s lagging or you’re having a hard time hearing the other person*,* that interrupts the intervention. That interrupts the mindfulness practice that you were just in the middle of and kind of taints the experience”* (ON, T3).

Disrupted continuity impacted client satisfaction, reducing desire to access services in this way. Participants described virtual counselling as *“totally disconnected”* (BC, T1). Lack of a personal connection to establish a rapport contributed to hesitation attending more intimate appointments over phone or video: *“I feel discouraged to go up to these online sessions. It still feels like you’re talking to the computer. It’s not the same”* (Ireland, T1).

For youth living with family or roommates, they felt the lack of privacy led them to be hesitant to access virtual services and inhibited from maximizing their therapeutic experience:

*“I wasn’t sure about [virtual services]… if I was able to do that in the home*,* because as I said before*,* like*,* everybody’s at home. It’s hard to find a space where it’s quiet and nobody can hear you. A space where you can really be vulnerable with the person that you’re talking to and trust that it’s only them that can hear the information and all that”* (Ontario, T1).

With regards to synchronous chat, the depersonalization aspect of texting was a concern due to inability to convey the full range of expression during diagnostic procedures: *“A lot of emotions and undertones aren’t really gotten across with text … from a diagnostic perspective*,* it’s probably not the best because people can really fake things via text*,* like*,* you could lie your way out of anything on text message*,* and nobody would know”* (ON, T1). For others, synchronous chat provided an alternative option when coping with anxiety due to flexibility of communicating in times of need without a scheduled appointment and no pressure: *“It’s not invasive or anything*,* and you can say as much as you want or stop replying when you want”* (Ireland, T1).

### Category 4. Mixed views on the pandemic over time: Attitudes, behaviour, and perception of policy response

This category summarizes how attitudes, behaviors, and perceptions of policy responses to the pandemic evolved over time among youth across the three recruitment locations. Initially, participants expressed mixed feelings about the pandemic, highlighting both concerns and adaptations in their daily lives. As time progressed, a shift in perceptions emerged, with some individuals recognizing the necessity of certain policies and their effectiveness, while others maintained skepticism about government responses. The way in which guidelines were poorly communicated to youth during the pandemic shaped youth in both countries in a similar way, leading to a nuanced interpretation of adhering and accepting the measures.

### Adherence to guidelines

Notable observations were made regarding how youth and other generations reacted to the pandemic over time. Fear of the unknown meant a greater willingness to follow public health guidelines such as physical distancing, wearing a mask, and frequently using hand sanitizer in the early days of the pandemic:

*“I think initially everyone was [taking it seriously]*,* young and old*,* were petrified*,* when we didn’t know. I think fear of the unknown at the start was why the first lockdown worked so well. Because everyone was really adhering*,* because like*,* no one knew how it [COVID-19] was going to affect you*,* you’d see the awful things on the TV and you didn’t know that sometimes you could literally have nothing [no symptoms]”* (Ireland, T1).

Others expressed willingness to adhere to public health guidelines in service of collective responsibility: *“I think that their role*,* like our role*,* everybody’s role is to try and stop the spread. So everybody has a responsibility to limit their contact”* (Ireland, T1).

Several youth expressed discontent regarding others not taking guidelines seriously, impacting how youth perceived the longevity of the pandemic: *“I also do see people around me that are not taking the pandemic very seriously … I just have frustrations with the current situation that make me not very optimistic about it”* (BC, T1). Social media became a space where youth could check in on what their peers were doing, influencing feelings of hope during this time: *“I see a lot of Instagram posts*,* social media posts where people are all together without a mask on*,* without anything*,* large group of people. That definitely brings down the hope a little bit … Situations like that can really set back the progress that’s been made”* (ON, T1).

Youth perceived their peer groups as adhering to guidelines more than older generations in terms of physical distancing: “*I feel like it’s usually the middle-aged people who are more not following the policies and rules of outdoor places*,* or even restaurants and whatnot. I think that for the most part*,* youth are being pretty respectful of that”* (BC, T1). Potential reasons for this difference were explored:

*“I think that teenagers as a whole have actually handled it very*,* very well. Because we’re so young*,* we’re like a sponge. We soak things up. We’re still being taught things. We’re still being told how to do things*,* as where*,* somebody in their 30s are kind of set in their ways”* (Ireland, T1).

Over time, youth demonstrated a declining adherence to public health guidelines, as one participant articulated: “*The fed-upness has taken over the wanting to be safe*” (Ireland, T3). Participants conveyed a sense of fatigue stemming from the frequency of lockdowns and the continually evolving nature of public health directives. Additionally, they noted a lack of targeted communication over time aimed at youth, which hampered their understanding of these changes and the shifting objectives associated with them.

*“I think*,* in the beginning we all saw how serious it was and we all kind of knew it was our*,* not job*,* but aim*,* to keep other people safe*,* so that’s why we were sticking to all the guidelines and we were doing what we were told and things like that. But in the past three months*,* being stuck at home*,* back lockdown again*,* school. I’ve seen a lot of people*,* and it shocked me*,* that have just completely given up on it*,* and just don’t want to do it anymore … I think they’ve kind of given up hope a little bit”* (Ireland, T2).

In this context, youth from all three geographical locations and across various genders articulated that the observed change in behavior should not be interpreted as a lack of concern. Rather, they attributed this shift to the cumulative effects of the pandemic on lifestyle changes and mental health. Participants noted that the escalating impact of these factors contributed to a growing sense of indifference toward compliance with established guidelines in each country. Despite the disillusionment with policy guidelines, increased anxiety about getting COVID-19 was also noted over time, across participants of all ages, genders, and geographical locations:

*“I think I’m more actively worried than I was previously. Before*,* I just kind of took my precautions – I did my best to stay out and that kind of thing. But now*,* I find myself getting stressed out at the grocery store when people are too near me. And I find myself actively avoiding certain situations*,* even more so than I did before … So it’s just – I feel a lot more anxious*,* right now*,* when I’m out in public”* (BC, T2).

Such concerns were worsened by the rise of new strains of COVID-19, reigniting fear of uncertainty of the severity of the disease and risk of a greater spread: *“Everyone now is talking about the Delta variant and it’s just so mentally draining”* (ON, T3). These fears led to participants experiencing a greater sense of hopelessness due to concern that the current resources to combat COVID-19 would not be effective long-term:

*“Just hearing that it is more deadly and more contagious*,* is definitely a worry. Because I know before people were thinking*,* well*,* you know*,* there are the vaccines coming out*,* so once I get it I’ll be fine. But you know*,* the findings that maybe it doesn’t work completely against the variants*,* that kind of makes it seem like there is no solution in place”* (ON, T2).

### Perception of policy response

Participants had mixed reactions to government policy response in all three regions. In Canada, the Canada Emergency Response Benefit (CERB) program, which provided financial support to Canadians whose employment income was affected by COVID-19, was viewed overwhelmingly as having a positive impact by alleviating financial strain. For students in college and university, having the financial support allowed youth to focus on their education and limit additional stressors:

*“I think the government definitely did help*,* especially with CERB cheques and things like that … having the government step in and financially assist me during this time was hugely beneficial*,* and allowing me to continue with my education*,* and not be stressed about money”* (BC, T1).

On the other hand, some participants in Ireland felt the COVID-19 Pandemic Unemployment Payments (PUP) were a source of stress, with some young person worrying *“that they’re going to make everyone pay back for COVID pay in tax”* (Ireland T3). Others felt frustrated by *“COVID pay”* (Ireland T3), causing tensions between those who received it and those who did not: *“My friend signed on to the COVID pandemic payment … I stopped talking to her for about four days*,* I was like … ‘I have to do 20 hours in three days. I am exhausted and you’re rubbing it in my face’”* (Ireland, T2).

While youth expressed contentment with restrictions implemented to keep people safe, a number of concerns were also raised. Over time, dissatisfaction centred around issues of changing guidelines and continuous lockdowns put in place by each region’s government: *“I think the way they’re going about it*,* as in a little-by-little*,* and then they open it a little bit and then they go back again*,* think that’s doing a lot of damage to people’s mental health”* (ON, T2).

### Absent youth voice in policymaking

A growing concern raised across sites and timeframes was a consistent lack of youth voice. Youth did not see themselves reflected in policy or feel they had an impact to steer outcomes, though they noted the importance of their voice for realising change for the youth population. In terms of policy decisions, youth expressed discontent with *“no youth at that table”* (BC, T1) and felt *“brushed to the side”* (Ireland, T3), describing, *“I feel like we have no say and we’re just following what other people are telling us to do”* (ON, T2). Lack of attention to youth issues contributed to a sense of invisibility: *“It’s like we don’t exist”* (Ireland, T2).

Despite desire to be involved in government and decision-making processes, youth noted minimal opportunity to engage: *“To a person who is young*,* them being able to put their voices out*,* that ability is very much hampered down*,* just because of these systemic barriers that are happening*,* and forces that work against young people”* (BC, T1). The absence of youth participation was most apparent concerning decisions around education. Many felt dissatisfied with schools closing and re-opening:

*“Being a student is unique*,* because I feel like most students were placed in a position where they didn’t really have a lot of power and we felt really passive in how we were receiving information and how decisions were being made in terms of our education*” (ON, T3).

As the last group to get the vaccine, youth raised concerns about returning to school: *“My university has mentioned that we would be opening up in September [2021] despite the fact basically no one would be up for vaccination until October at the earliest. I feel like that’s something someone could have pointed out”* (ON, T2). Lack of choice regarding decisions to continue school virtually or in-person created strife among university-aged students: *“Going back to campus full time without any online options*,* I don’t think that a young person would necessarily make that decision”* (BC, T3). Frustration continued to grow over time as the perception of neglect persisted: *“There’s been such a lack of concern for university age people*,* you know? That I think people are getting a bit disgruntled”* (Ireland, T3).

Youth recognized the value in having their opinion shared despite being young: *“Just because you’re 20 doesn’t mean your experience is worth less”* (BC, T3). Desire for involvement stemmed from an understanding of being future decision makers: *“We’ll be the next people in-charge of things. Every generation can build on the last generation … I think that’s what makes young people in the world so important*,* because we can all learn from people’s past mistakes and build on that and stuff”* (Ireland, T2). With this mindset, youth described the importance of engaging in activism and online petitions and voting when of age in order to effect change.

### Unfair judgement of youth role in the pandemic

A recurring sentiment among participants was the perception that youth were being disproportionately blamed for the spread of infection in the media. Youth described feeling *“unfairly targeted”* (BC, T1) upon experiencing criticism for breaking rules and not taking the pandemic seriously:

*“We’ve all been labelled like this*,* you know*,* ‘it’s the young spreading the virus’*,* ‘it’s the young that don’t care’*,* but it’s not like that*,* that’s not true*,* like I’m taking all this very serious. It’s affected my life in ways that I could never have even imagined”* (Ireland, T1).

In light of these accusations, youth countered such blame by pointing out how they are vulnerable to infection as the last age group to be eligible for the vaccine, as well as a demographic notably on the front lines of customer service and hospitality jobs, placing them at increased risk compared to those with the option to work from home:

*“… [Young people] are sort of being blamed for a lot of the spread*,* but also*,* that age category is probably a lot of the people who’re working minimum-wage*,* front-line jobs*,* being forced to be out in places*,* taking public transit*,* living with roommates … And we’re going to be the last to be vaccinated which is*,* again*,* frustrating”* (ON, T2).

### Vaccination in the face of hesitation

Overall, youth expressed willingness to get a COVID-19 vaccine. For those with concerns, hesitancy was raised surrounding uncertainty about the speed at which the vaccines were developed and rolled out in addition to not knowing the long-term impacts. Such concerns were most commonly raised at T1, prior to vaccines becoming available: *“I’m not anti-vaccine*,* but I feel like they are rushed … You never know what the side effects are in a year or ten years from that vaccine*,* right? That’s something that I worry about”* (BC, T1). Some youth felt like the misinformation online contributed to their uncertainty about the vaccine:

*“I have mixed feelings about [the vaccine] … I feel like I feed in to Facebook been like*,* oh*,* it will make you have a brain damage … don’t take it*,* [you’ll] never have children and stuff like that. But I feel like if it was that bad*,* they wouldn’t be giving it to people. So*,* I just look at actual scientific side of it rather than feed into random people on Facebook”* (Ireland, T2).

To address these concerns, youth described needing more information, presented in a way that explains how the vaccine works:

*“I want to know information and I want to learn about it. I wouldn’t let anyone put any vaccine into me without knowing about it. So*,* I think what has really worried me is that the information and the responses have been so*,* so vague to a point where it seems really shady”* (ON, T2).

Despite hesitation, youth discussed several motivating factors that led them to get a COVID-19 vaccine, primarily for functional reasons and as a means to return to normalcy by T2 and T3. Youth noted how a vaccine would provide a sense of safety: *“I am at ease that something is protecting me”* (ON, T3). More than just in a practical sense, vaccines gave participants a renewed sense of optimism:

*“What is exciting about the vaccine is the potential to dream about the future again in a meaningful capacity. In a way*,* COVID took away the ability to conceive of any future because the future became indistinct*,* right. But having the vaccine allows you to go back to imagining a future that is with other people which is important”* (ON, T2).

### Category 5. Navigating COVID-19 information

In this category, participants reported the complexity involved with navigating COVID-19 information and the associated confusion with content posted on social networks, news articles, and within their own personal networks. Youth across all three countries relied on social media and online platforms for updates, which sometimes led to misinformation and uncertainty, and an overall feeling that the content was not developed for youth by youth. To counter this, many actively sought reliable information from trusted sources, such as health organizations and educational institutions. Peer discussions, primarily online through platforms such as TikTok, also played a crucial role in shaping their understanding, as youth shared insights and strategies to cope with the evolving situation. Youth emphasized the need to develop their own approach to critically engage with information, despite the challenges posed by the rapidly changing landscape of the pandemic.

### Confusion around pandemic information presented by the government

Youth found pandemic information confusing and containing too much jargon: *“It was not easy to understand. Just a lot of big words and government words”* (BC, T1). As a result of inaccessible language, youth were deterred from seeking out information: *“I don’t listen to it anymore because it’s too confusing*,* it’s too much information being thrown out in 5-minute speeches”* (Ireland, T1). Over time, as guidelines changed, participants felt it was hard to follow and keep up to date with current guidelines: *“I think maybe the government could’ve done a bit better of mitigating some of that ambiguity and uncertainty*,* and helping people cope with the ever-changing guidelines and protocols”* (BC, T1). The need for youth-friendly information was addressed:

*“I feel like they should have like some younger people on the government’s team just to like explain what some things mean to the younger generations … me and some of my friends have read some stuff from the government website*,* and we’re just like*,* ‘what does even half of this mean?’”* (ON, T1).

### Accessing COVID-19 information through multiple forms of media

The primary sources where youth accessed pandemic information were the government and news media’s social media, webpages, and daily addresses. Having options tailored to the needs of the individual was beneficial:

*“I really like the Prime Minister’s address …because I have dyslexia*,* I don’t really like reading*,* I don’t like going through long articles. I need to just hear it … So*,* I just appreciated someone putting everything into a concise press conference so I don’t have to go hunting for all the articles*,* hunting for the information and everything”* (ON, T1).

Social media was commonly discussed, as participants described using various platforms such as verified news outlets and government run Instagram, TikTok, Twitter, Reddit, and YouTube to access information from people or organizations whom they trust:

*“I think the government actually did a decent job of putting things on Instagram. I wasn’t really active on any other platform*,* but just seeing like*,* you know*,* the infographics about*,* here’s what you’re allowed to do”* (BC, T3).

Youth were astutely aware of the need to find reliable sources and wade through misinformation, citing the spread of false information as a barrier to staying informed:

*“It’s very hard to avoid any kind of misinformation or just complete lies about it. That’s kind of a difficult thing*,* and I’d say that’s where a lot of difficulty in miscommunication comes from is just because people’s primary new source is the internet*,* which is not always very reliable”* (Ireland, T3).

### Category 6. Transitioning to life after the pandemic

This category summarized how youth across the two countries experienced the complex transition back to a “new normal”. This transition reflected fear, resilience and adaptability, as young people worked to rebuild their lives in a changed world. This is described in more detail in three sub-categories described in detail below.

### Cautious readjustment to a new normal

Young people anticipated challenges with re-adjusting to life after the pandemic with years of diminished social interaction and physical distancing, particularly felt in the midst of lockdowns: *“Everyone’s afraid of each other”* (Ireland, T2). Participants described a persistent cautious outlook when attending public events or gatherings, with one participant saying, *“I think immediately after the pandemic*,* there’s going to be a lot of people who are a little skittish about re-integration into crowds and restaurants”* (ON, T2). Such challenges with reintegration were recognized *“as almost a trauma response*,* where if someone gets too close to you*,* you feel unsafe”* (BC, T1).

Some participants grew comfortable with spending time alone, with long-lasting impacts on their desire for socialization: *“I’ve gotten used to my own company and I’m starting to enjoy just being with myself but that’s also a negative”* (Ireland, T2). Participants recognized the importance of social interaction, though felt as though transitioning from spending time alone to engaging with others in person might be challenging: *“I feel like it’s going to be hard to go back to real life*,* because I’m so used to not leaving my house*,* now*,* and I’m kind of like a bear in hibernation*,* now. I don’t know if that’s going to be an easy transition”* (BC, T1). Balancing the needs for social interaction with the comfort of spending time alone was thought to be a difficult balance to navigate, with concerns for long-term mental health impacts. Participants recognized these challenges as collective trauma: *“Some people will be traumatized by it and may never heal from it”* (ON, T2). Without adequate supports in place, youth feared for their ability to heal moving forward.

### Concerns for the future

When considering their futures in a post-pandemic world, participants expressed concern for the state of the economy as well as their own job prospects: *“I think economically Ireland will have a long time recovering and especially for when I’m older and I’m buying a house. I feel like the prices will still be really high”* (Ireland, T1). In light of these concerns, several youth noted that they re-evaluated their plans and goals for the future to adjust to a new world and changes to their own self, considering, *“What is post-COVID for me now*,* because I am in a much different place than I thought I would be”* (ON, T3). In light of these fears, youth described the need for support, though had concerns that their needs would not be met, particularly for those without a strong social support network:

*“I do think [young people] face unique circumstances and I think because of a lot of income inequality*,* like the general expectations for new graduates*,* I think young people have a lot of challenges that have been exacerbated by the pandemic*,* and I feel like if there aren’t the correct social safety nets in place. It can leave a lot of young people behind … You know*,* not everyone has a family that they can rely upon during these sorts of times*,* and I think we just have to make sure that we’re looking out for everyone”* (BC, T1).

### Optimism about positive social changes

Despite hardships endured during the pandemic, youth were able to identify unseen benefits and potentially positive long-term changes in terms of the workplace, education, hygienic practices, and social justice. Youth hoped to see flexibility regarding work-from-home policies, *“where workplaces are more lenient*,* [and] people understand things like childcare”* (BC, T1), with improved accommodations for employee needs: *“I think more people are going to be opting to work from home if they’re allowed to because commuting to work doesn’t really do much for them”* (ON, T2).

Youth were optimistic about improved health beyond the pandemic. Continued hygienic practices to prevent spread of illness beyond COVID-19 were discussed in a positive light: *“Sanitizing*,* washing our hands*,* wearing masks when we’re sick*,* taking time off to stay at home when we’re sick*,* I feel like those are going to have long-term affects within the workplace*,* and within school”* (BC, T2). While exacerbated mental health challenges were noted as a continued uphill battle to address, the pandemic sparked more conversation around mental health, trauma, and help-seeking that resulted in de-stigmatization, which was thought to be a step in the right direction:

*“I hope in the recovery of Ireland and the economy of Ireland and stuff*,* they don’t forget about mental health because it was such an important thing during COVID and I hope that’s*,* that slowly doesn’t fade away” (Ireland*,* T1).*

Because of the political discourse throughout the pandemic with attention to social justice issues, youth were optimistic that some of the structural inequities brought to light would be addressed in society: *“I think that will be the big frontier*,* because I think the pandemic*,* for all the bad*,* does provide an opportunity to rectify some of the inequalities we’ve seen exposed through it”* (BC, T1). Inherently tied to these changes was a developed sense of compassion and empathy among youth and others, and the belief that change is possible:

*“I’m hoping this period helps develop empathy and kindness in people … I’m hoping that this is an opportunity for people to kind of reflect on the skills that they offer in that sense and hopefully grow in that sense*,* and using those newfound skills and newfound parts of themselves to engage with their communities and to take care of their communities*,* whether that’s physically or by leading it on a kind of social change front or whatever that may look like”* (ON, T1).

In this sense, youth expressed actionable strategies to use their voice through social media and partaking in social justice movements to pressure policymakers: “*We deserve health and equity*,* and we have the resources and the capability to have those things*,* we just need to make it happen”* (ON, T3). Participants found hope in this belief, with the change starting within themselves: *“I know me*,* for one*,* I’ve come out of it a better person”* (Ireland, T2).

### Category 7. Youth-led recommendations for government and service response

Four subcategories about key recommendations for government and service response were generated from the data over time, which related to strategies for amplifying youth voice, communicating public health information effectively to youth and combatting misinformation, optimizing services post-pandemic, and planning for future pandemics.

### Youth voice needed in decision-making

Participants raised the importance of incorporating youth voices in policy decisions, particularly when directly impacted by the outcome. Youth articulated the need for their voices to shine through research so that policymakers understand their needs, with attention to questions such as, *“How are you living your life? What are your priorities? What are your concerns?”* (ON, T3). School was recognized as the best way to reach a broad spectrum of youth: *“I feel like the only way you could really collect information from a bunch of young people is through school”* (ON, T3).

In addition to research, youth expressed desire for more opportunities for leadership to develop skills in advocacy and bridge the gap between youth and those in charge. Participants noted that adults in positions of power have an important role in creating such opportunities, where leaders *“who have paved the way for other youth to use their voice [are] really important”* (BC, T1). Opportunities at school for youth to address issues were discussed: *“I feel like there could be maybe like a group of students and young people who can kind of just voice the opinions of everyone on their behalf and stuff and maybe just offer up suggestions”* (Ireland, T1). On a broader level, the establishment of a youth advocate role within government was suggested as a means to effectively communicate the specific needs of young people, rather than relying on assumptions made by adults who may not fully understand their perspectives (Ireland, T1, BC T3).

The importance of diversity in youth experiences was recognized, with understanding that historically, persistently, or systemically marginalized youth with important stories to share may be ostracized from participation in research and decision-making:

*“I feel like there are definitely people who should be involved but the people who should be involved are not usually the people who can be involved. Like if you are poor and in an immigrant household you are probably at home taking care of you siblings and have no time to sit on committee meetings”* (ON, T2).

### Communicating public health information effectively to youth and combatting misinformation

Youth discussed the need to continue to *“use social media in a way that you promote responsible information or reliable information”* (ON, T3) because *“that’s where a lot of young people spend a lot of their time”* (BC, T3). Concise, digestible content in the form of infographics was said to be an effective way to disseminate information, promote services, and encourage service uptake: *“None of the BS*,* you know … cut all the medical jargon out … explain it simply”* (Ireland, T1).

With regard to sharing COVID-19 information, youth thought it *“would be helpful if there was more clarity as to why each restriction was put in place”* (Ireland, T1) to combat frustrations with fluctuating and sometimes contradictory restrictions and to improve adherence to guidelines.

“I think it would be helpful if there was more kind of like clarity as to why each restriction is put in place, because people would be like ‘Hang on. So we can’t do this, but people can like, come into the country and leave the country.’ And like ‘We can do this at school, but we can’t do this in shops’. And they’ll be like, why is that? And they kind of get frustrated and be like, I’m not listening to them anymore. So I think it would be helpful if there is more thorough explanations as to why, like maybe you can do this, but not that, and stuff. " [el].

Aiming information at parents is another way to spark dialogue between parents and their children about mental health: *“I think by targeting adults*,* that would help the situation for younger people to feel more comfortable talking about their issues”* (BC, T3).

Aside from social media, school was reiterated as a means to convey information to youth, teach emotional literacy, and provide resources: *“I think getting information to students through institutions; I think it’s a very smart move. Like keeping up with your school’s newsletter”* (ON, T3).

Creating visibility surrounding resources and mental health can promote help-seeking, as recognized by a university student in BC: *“I feel like sometimes it’s like you have to go out and seek it*,* but it’d be nice if universities sent out emails midway through the semester*,* just to tell people about that”* (BC, T2).

A key issue raised by participants regarding accessibility of virtual information was the prevalence of misinformation and knowing how to identify reliable sources. As a generation who grew up with social media, the need for strategies to address these issues was apparent. Youth expressed desire to learn about internet literacy and critical thinking skills in the classroom:

*“I think internet literacy*,* where if people grow up and they learn*,* OK*,* not everything on the internet is true … If that thought process was taught in school*,* then it wouldn’t really be as big a problem as it is today”* (ON, T2).

Similarly, accountability within the platforms themselves and in government was suggested as a welcomed solution:

*“I think holding the people accountable that spread misinformation will help and there being less bots or problematic ideas going … tech companies holding people accountable*,* or governments holding people accountable”* (ON, T3).

### Post-pandemic services: enhancing service delivery to improve outcomes

An issue brought to light during the pandemic was the lack of affordable and accessible mental health services, worsened by intensifying mental health problems experienced during this time. For youth, the pandemic presented an opportunity to address some of the issues faced within the system that impact youth specifically. Despite a desire to seek help, lack of affordable and timely services created a major barrier to care:

*“A lot of people want mental health services and either they can’t afford it or they’re on like a six-month*,* one-year waiting list”* (ON, T3).

To address this issue, youth recommended expanding social safety nets to cater to the needs of this population:

*“Starting with the expansion of disability benefits*,* making it so that people who are on disability are not living in poverty*,* that would be a good start*,* because we are going to have a fall out in terms of the people who have been affected by COVID”* (ON, T3).

In the aftermath of COVID-19, there was an expressed desire for priority to be given to mental health services to counterbalance the mental health challenges that were worsened by increased stress and decreased access to services:

*“The government’s definitely going to have to implement some sort of strategy*,* like for aftercare*,* I feel. I just feel like it’s going to be a domino effect and it’s going to go on for years to come … You just don’t know what kind of effect that would have on somebody”* (Ireland, T2).

Post-pandemic services should also continue to offer virtual care according to participants. While many youth declared a preference for in-person services, there was recognition of the value in having options:

*“[Services should] still be definitely available online*,* especially because some people*,* you know*,* maybe aren’t comfortable [with] the face-to-face environment yet*,* or maybe are immunocompromised or just physically can’t get there”* (BC, T3).

### Planning for future pandemics

In a future pandemic, youth emphasized that personalizing messaging to all demographics is important. In the youth context, this can be seen as communicating information regarding an infectious disease in a way that is easy to understand, digestible, and does not instill fear and prioritizes facts:

*“I really like simplicity*,* like getting to the point. Easy facts. Anything factual information that has key points and bolded and have diagrams*,* pictures*,* or even*,* like*,* an example*,* analogies*,* et cetera*,* would be really important to understand”* (BC, T1).

Ensuring resources and funding are available would help youth feel supported in times of crisis. Not all youth have strong familial relationships to provide safety and protection, requiring implementation of safety nets:

*“Far too often*,* the young-person demographic*,* so like not a minor*,* but not a middle-aged person*,* is often ignored in planning stages. It’s assumed that we can operate as those people have*,* but I think we have a much less robust support system*,* and even just crisis funds and things like that. I think that needs to be accounted for”* (ON, T2).

In light of these recommendations, it is evident that the COVID-19 pandemic constitutes not merely an acute public health crisis but a historical event with enduring implications for youth health and wellness for many years to come. The pandemic has revealed significant gaps in service delivery for this population, highlighting the necessity for a comprehensive approach to address their ongoing needs, ideally with diverse youth at the centre of all decision making, knowledge sharing, and future planning.

## Discussion

This study examined youth experiences during COVID-19 over an extended period, providing valuable, youth-generated recommendations for government and service responses to pandemics and other public health emergencies. Our longitudinal qualitative analysis revealed critical trends that demonstrate the multifaceted impact of COVID-19 on participants at various stages of the pandemic, with consistent findings across regions, including BC and Ontario in Canada, as well as Ireland. These insights underscore the necessity of incorporating youth perspectives into future public health strategies. Youth described their experiences of coping with the many hardships of the pandemic and adapting to new ways of accessing services, but they also identified areas for personal growth through the hardship. Challenges with transitioning to a post-pandemic life were similar across both countries. Youth leveraged their experiences to provide recommendations for responses by governments and service-providing organizations for future pandemics and public health emergencies.

Findings were consistent at each time point, despite some small differences in the cohorts. This may be due to the similar COVID-19 responses in these regions, including lockdown measures, vaccination rollout, testing, and contact tracing [40]. Their adaptive responses all included a series of lockdowns comprising of stay-at-home orders, school and non-essential business closures, a vaccination strategy that prioritized vulnerable populations and essential workers, mandatory use of face coverings, and providing income support to affected workers [40]. Furthermore, ON, BC, and Ireland have integrated youth services that provided virtual mental health services to youth throughout the pandemic [30, 35, 41]. While Ireland had more confirmed COVID-19 cases per capita than ON and BC, this did not seem to influence the impacts on youth [42]. The sample of youth from Ireland were younger on average, and more focus on school and family was found in the data. These findings align with observations in similar populations in other high-income countries [40]. Nevertheless, it is crucial to acknowledge that further research on the impacts of the COVID-19 pandemic on youth in areas with lower income, a varied COVID-19 response, and/or without integrated youth services would be beneficial to assess the applicability of these findings across wider regions.

The challenges encountered in response to the COVID-19 pandemic are multifaceted and align with emerging literature. For instance, the loss of significant life experiences has been identified as a critical factor impacting young people in profound ways during the pandemic [43, 44]. Additionally, educational challenges arose, particularly in the form of difficulties adjusting to online learning environments [24, 45–47]. Job loss and financial instability contributed to cumulative stress over time, driven by sudden economic disruptions and uncertainty about the future [48, 49]. Essential workers faced heightened risks upon returning to work, grappling with the dual pressures of potential COVID-19 exposure and the new responsibilities associated with enforcing public health guidelines [50]. Furthermore, there was a notable increase in incidents of racism and xenophobia, compounding the adversities faced by many individuals. Anti-Asian attitudes in the age of COVID-19 have been documented in the American context [51–53], noting higher rates of harassment and health disparities throughout the pandemic due to harmful rhetoric regarding COVID-19 origins. Difficult-to-follow public health information was an additional challenge, as was initial vaccine hesitancy [54–58] (Everest L, Henderson J, Prebeg M, Relihan J, Ma C, Hawke LD: Relationship between mental health on COVID-19 vaccine hesitancy in youth: a mixed methods longitudinal cohort study, Under review), although attitudes shifted toward favouring vaccines with time. With the myriad of challenges came a decline in mental health and mixed effects on substance use behaviors, particularly noted in T1 and T2, as is documented in the literature cross-sectionally and longitudinally [16–20, 29, 59–61].

With the complex changes in contexts, environments, and lifestyle, youth also experienced disruptions in health service accessibility, including mental health service access. These disruptions may have further constituted a barrier to health and wellness [31, 62], although acceptance of virtual services seemed to increase with time. Isolation, financial strain, and setbacks in educational and professional development in the context of reduced service accessibility may be contextual factors that led to the short-term impacts on mental health; this trajectory could also extend into long-term impacts on mental health and substance use, which is an important area for ongoing research as the pandemic resolves [63].

Despite the hardships experienced, youth also identified several strengths and opportunities for growth during the COVID-19 pandemic. Youth in both countries described picking up new skills and hobbies such as cooking, exercise, and creative endeavors, which helped with overall wellness. In some cases, mental health improved due to reduced demands on time and increased time to self-reflect, slow down, and recover from burnout. In this sense, the pandemic was a catalyst for change where help-seeking and engagement in services were realized for the first time. Access to green spaces was a positive factor; indeed, this has been shown to reduce symptoms of depression, anxiety, and stress [64–66]. A small body of research discusses the positive developmental opportunities, strengths, and effective coping strategies leveraged by youth during the changes in the pandemic [8, 67]. Another body of research outlines post-traumatic growth in various populations during this time [67, 68]. Together, these findings can serve to identify strengths, protective factors, and resiliencies that can be harnessed to support mental health and wellbeing during challenging periods in history, from a pragmatic and strengths-based lens. Indeed, youth remained optimistic about the potential for the pandemic to act as a catalyst for positive social change.

Youth expressed concerns regarding the insufficient attention given to their needs, the unique adversities they faced, and their lack of engagement and leadership in formulating solutions during the pandemic response. Consistent with existing literature [69, 70], it is imperative to prioritize youth involvement in research and policymaking by centering their issues and recognizing them as equal stakeholders and rights holders. The Children and Youth in Challenging Contexts (CYCC) Network report (2013) outlines best practices for engaging youth to combat social exclusion and mitigate power imbalances, emphasizing the necessity of collaboration [71]. Organizations, educational institutions, and governmental bodies at all levels should enhance their capacity to create platforms that enable youth to voice their concerns on pertinent issues and ensure that their perspectives are actively considered. Establishing student and youth advocacy groups, along with dedicated government liaison positions, can facilitate the bridging of gaps and foster increased political participation among young people.

Based on the current findings and the emerging literature, examples of solutions that youth might propose to better address future pandemics and public health emergencies include the use of verified, fact-checked social media posts to clearly communicate public health information in a consistent manner [72, 73], together with strategies to eliminate help-seeking barriers and support system navigation [74, 75]. Incorporating age-appropriate internet literacy into school curricula to help cultivate critical thinking skills and discern factual information from unreliable sources was also discussed. Free or affordable mental health services are needed, as well as attention to accessibility needs. Attention should be given to enhancing access for those with minimal technological literacy skills [76]. Financial supports and means to safely socialize, exercise, and alleviate symptoms of mental illness are additional youth priorities. In a similar vein, resources should be distributed to address safety concerns for those in unsafe households, who may be at risk of emotional or physical abuse during periods of lockdown. Additional novel goals and solutions may be proposed through ongoing and appropriate youth engagement in policy, service, and research design.

### Strengths and limitations

This study provided longitudinal insights that capture a substantial period of change during the evolving COVID-19 pandemic. Spanning multiple jurisdictions with large samples, the study identified similar cross-cutting experiences reported by a wide variety of youth. With youth engagement throughout, the research questions and approaches were informed by youth voices. However, limitations of this study include response bias, varied sampling strategies and timelines across study sites, complicating comparisons between sites, as well as attrition. While flexibility in sampling strategies is a strength of qualitative longitudinal research design for reasons including enhancing generalizability of findings, comparability can also be a limitation. The variability in recruitment approaches across different sites may have hindered the ability to discern consistent patterns and trends over time. Future research is needed to distinguish patterns amongst different groups (e.g., gender, age, race/ethnicity). Given the longitudinal nature of this study, participant numbers declined over time. As the findings suggest, “COVID fatigue” may have contributed to study drop-out. Those who chose to remain in the study may differ from those who did not complete all three timelines. The use of virtual means to recruit and conduct the interviews expanded the geographical reach of the study, but limited it to youth with online access.

## Conclusion

This is the first known international longitudinal qualitative study to describe youth experiences during the COVID-19 pandemic across two Canadian jurisdictions and Ireland, with implications for policy and practice. Despite the many challenges encountered over the course of the pandemic, youth reported aspects of growth. For this population, pre-existing stressors were compounded by pandemic-related challenges and reduced access to care, signifying the importance of prioritizing youth mental health moving forward. Areas of need highlighted throughout the pandemic have implications for future policy decisions, with an opportunity to enhance health and social service delivery. Investment in resources rooted in youth-identified needs and priorities is vital to mitigate the long-term challenges associated with pandemic trauma among youth.

## Supplementary Information

Supplementary Material 1.

## Data Availability

Due to multiple sites participating, the data sets generated during the current study are not publicly available but are available from the corresponding author under reasonable request.

## References

[1] Mesman E, Vreeker A, Hillegers M. Resilience and mental health in children and adolescents: an update of the recent literature and future directions. Curr Opin Psychiatry. 2021;34:586–92. 34433193 10.1097/YCO.0000000000000741 PMC8500371

[2] Callahan KL, Hilsenroth MJ, Yonai T, Waehler CA. Longitudinal stress responses to the 9/11 terrorist attacks in a New York metropolitan college sample. Stress Trauma Crisis. 2005;8:45–60. 10.1080/15434610590913621.

[3] Li J, Zweig KC, Brackbill RM, Farfel MR, Cone JE. Comorbidity amplifies the effects of post-9/11 posttraumatic stress disorder trajectories on health-related quality of life. Qual Life Res. 2018;27:651–60. 10.1007/s11136-017-1764-5. 29260446 10.1007/s11136-017-1764-5 PMC5845593

[4] Neria Y, DiGrande L, Adams BG. Posttraumatic stress disorder following the September 11, 2001, terrorist attacks: a review of the literature among highly exposed populations. Am Psychol. 2011;66:429–46. 10.1037/a0024791. 21823772 10.1037/a0024791 PMC3386850

[5] Roberts YH, Mitchell MJ, Witman M, Taffaro C. Mental health symptoms in youth affected by Hurricane Katrina. Prof Psychol Res Pr. 2010;41:10–8. 10.1037/a0018339.

[6] Weems CF, Taylor LK, Cannon MF, Marino RC, Romano DM, Scott BG, et al. Post traumatic stress, context, and the lingering effects of the Hurricane Katrina disaster among ethnic minority youth. J Abnorm Child Psychol. 2010;38:49–56. 10.1007/s10802-009-9352-y. 19707864 10.1007/s10802-009-9352-y

[7] United Nations Department of Economic and Social Affairs. Definition of youth. United Nations Inter-Agency Network on Youth Development. https://www.un.org/esa/socdev/documents/youth/fact-sheets/youth-definition.pdf.

[8] Hawke LD, Daley M, Relihan J, Semansky P, Sheth MS. Reaching youth with reliable information during the COVID-19 pandemic: Social media for sure. Int J Child Youth Fam Stud. 2023;14(3):1–21. 10.18357/ijcyfs143202321632.

[9] Hawke LD, Sheikhan NY, MacCon K, Henderson J. Going virtual: Youth attitudes toward and experiences of virtual mental health and substance use services during the COVID-19 pandemic. BMC Health Serv Res. 2021;21:340. 10.1186/s12913-021-06321-7. 33853602 10.1186/s12913-021-06321-7 PMC8045568

[10] Al Omari O, Al Sabei S, Al Rawajfah O, Abu Sharour L, Aljohani K, Alomari K, et al. Prevalence and predictors of depression, anxiety, and stress among youth at the time of COVID-19: an online cross-sectional multicountry study. Depress Res Treat. 2020;2020:1–9. 10.1155/2020/8887727. 10.1155/2020/8887727 PMC7537692 33062331

[11] Blackwell CK, Mansolf M, Sherlock P, Ganiban J, Hofheimer JA, Barone CJ, et al. Youth well-being during the COVID-19 pandemic. Pediatrics. 2022;149. 10.1542/peds.2021-054754. 10.1542/peds.2021-054754 PMC9169239 35301542

[12] Liang L, Ren H, Cao R, Hu Y, Qin Z, Li C, et al. The effect of COVID-19 on youth mental health. Psychiatr Q. 2020;91:841–52. 10.1007/s11126-020-09744-3. 32319041 10.1007/s11126-020-09744-3 PMC7173777

[13] Courtney D, Watson P, Battaglia M, Mulsant BH, Szatmari P. COVID-19 impacts on child and youth anxiety and depression: challenges and opportunities. Can J Psychiatry. 2020;65:688–91. 10.1177/0706743720935646. 32567353 10.1177/0706743720935646 PMC7502880

[14] Prowse R, Sherratt F, Abizaid A, Gabrys RL, Hellemans KGC, Patterson ZR, et al. Coping with the COVID-19 pandemic: examining gender differences in stress and mental health among university students. Front Psychiatry. 2021;12. 10.3389/fpsyt.2021.650759. 10.3389/fpsyt.2021.650759 PMC8058407 33897499

[15] Quinlan-Davidson M, Shan D, Courtney D, Barbic S, Cleverley K, Hawke LD, et al. Associations over the COVID-19 pandemic period and the mental health and substance use of youth not in employment, education or training in Ontario, Canada: a longitudinal, cohort study. Child Adolesc Psychiatry Ment Health. 2023;17:105. 10.1186/s13034-023-00653-4. 37679811 10.1186/s13034-023-00653-4 PMC10486040

[16] Ertanir B, Kassis W, Garrote A. Longitudinal changes in Swiss adolescent’s mental health outcomes from before and during the COVID-19 pandemic. Int J Environ Res Public Health. 2021;18:12734. 10.3390/ijerph182312734. 34886458 10.3390/ijerph182312734 PMC8656984

[17] Hawes MT, Szenczy AK, Klein DN, Hajcak G, Nelson BD. Increases in depression and anxiety symptoms in adolescents and young adults during the COVID-19 pandemic. Psychol Med. 2022;52:3222–30. 10.1017/S0033291720005358. 33436120 10.1017/S0033291720005358 PMC7844180

[18] Layman HM, Thorisdottir IE, Halldorsdottir T, Sigfusdottir ID, Allegrante JP, Kristjansson AL. Substance use among youth during the COVID-19 pandemic: a systematic review. Curr Psychiatry Rep. 2022;24:307–24. 10.1007/s11920-022-01338-z. 35476186 10.1007/s11920-022-01338-z PMC9043089

[19] Hawke LD, Sheikhan NY, Oates S, Daley M, Dixon M, Henderson J. Impact potpourri: a Multimethod Survey Study on Youth Substance Use during COVID-19. Can J Addict. 2022;13:46–55. 10.1097/CXA.0000000000000158. 36452036 10.1097/CXA.0000000000000158 PMC9677386

[20] Thorisdottir IE, Asgeirsdottir BB, Kristjansson AL, Valdimarsdottir HB, Jonsdottir Tolgyes EM, Sigfusson J, et al. Depressive symptoms, mental wellbeing, and substance use among adolescents before and during the COVID-19 pandemic in Iceland: a longitudinal, population-based study. Lancet Psychiatry. 2021;8:663–72. 10.1016/S2215-0366(21)00156-5. 34090582 10.1016/S2215-0366(21)00156-5

[21] Atlam E-S, Ewis A, El-Raouf MMA, Ghoneim O, Gad I. A new approach in identifying the psychological impact of COVID-19 on university student’s academic performance. Alexandria Eng J. 2022;61:5223–33. 10.1016/j.aej.2021.10.046.

[22] Sahu P. Closure of universities due to coronavirus disease 2019 (COVID-19): impact on education and mental health of students and academic staff. Cureus. 2020. 10.7759/cureus.7541. 32377489 10.7759/cureus.7541 PMC7198094

[23] United Nations. Policy brief: Education during COVID-19 and beyond. 2020. https://www.un.org/development/desa/dspd/wp-content/uploads/sites/22/2020/08/sg_policy_brief_covid-19_and_education_august_2020.pdf.

[24] Nandlall N, Hawke LD, Hayes E, Darnay K, Daley M, Relihan J, et al. Learning through a pandemic: Youth experiences with Remote Learning during the COVID-19 pandemic. Sage Open. 2022;12:215824402211241. 10.1177/21582440221124122. 10.1177/21582440221124122 PMC9511001 36185703

[25] Deng Z, Arim R, Henseke G, Schoon I, Dietrich H, Murray A et al. Youth unemployment in Canada, Germany, Ireland, and the United Kingdom in times of COVID-19. 2022. Available: https://www150.statcan.gc.ca/n1/pub/36-28-0001/2022003/article/00003-eng.htm.

[26] Benner AD, Mistry RS. Child development during the COVID-19 pandemic through a life course theory lens. Child Dev Perspect. 2020;14:236–43. 10.1111/cdep.12387. 33230400 10.1111/cdep.12387 PMC7675461

[27] Wolf K, Schmitz J. Scoping review: longitudinal effects of the COVID-19 pandemic on child and adolescent mental health. Eur Child Adolesc Psychiatry. 2023. 10.1007/s00787-023-02206-8. 37081139 10.1007/s00787-023-02206-8 PMC10119016

[28] Buizza C, Bazzoli L, Ghilardi A. Changes in college students mental health and lifestyle during the COVID-19 pandemic: a systematic review of longitudinal studies. Adolesc Res Rev. 2022;7:537–50. 10.1007/s40894-022-00192-7. 35966832 10.1007/s40894-022-00192-7 PMC9362152

[29] Liu SR, Davis EP, Palma AM, Sandman CA, Glynn LM. The acute and persisting impact of COVID-19 on trajectories of adolescent depression: sex differences and social connectedness. J Affect Disord. 2022;299:246–55. 10.1016/j.jad.2021.11.030. 34798146 10.1016/j.jad.2021.11.030 PMC8605896

[30] McGorry PD, Mei C, Chanen A, Hodges C, Alvarez-Jimenez M, Killackey E. Designing and scaling up integrated youth mental health care. World Psychiatry. 2022;21:61–76. 35015367 10.1002/wps.20938 PMC8751571

[31] Hawke LD, Szatmari P, Cleverley K, Courtney D, Cheung A, Voineskos AN, et al. Youth in a pandemic: a longitudinal examination of youth mental health and substance use concerns during COVID-19. BMJ Open. 2021;11:e049209. 10.1136/bmjopen-2021-049209. 34716160 10.1136/bmjopen-2021-049209 PMC8561825

[32] Hawke LD, Koyama E, Henderson J. Cannabis use, other substance use, and co-occurring mental health concerns among youth presenting for substance use treatment services: sex and age differences. J Subst Abuse Treat. 2018;91:12–9. 10.1016/j.jsat.2018.05.001. 29910010 10.1016/j.jsat.2018.05.001

[33] Henderson JL, Brownlie EB, McMain S, Chaim G, Wolfe DA, Rush B, et al. Enhancing prevention and intervention for youth concurrent mental health and substance use disorders: the research and action for teens study. Early Interv Psychiatry. 2019;13:110–9. 10.1111/eip.12458. 28745011 10.1111/eip.12458 PMC6492445

[34] Henderson JL, Cheung A, Cleverley K, Chaim G, Moretti ME, de Oliveira C, et al. Integrated collaborative care teams to enhance service delivery to youth with mental health and substance use challenges: protocol for a pragmatic randomised controlled trial. BMJ Open. 2017;7:e014080. 10.1136/bmjopen-2016-014080. 28167747 10.1136/bmjopen-2016-014080 PMC5293997

[35] Mathias S, Tee K, Helfrich W, Gerty K, Chan G, Barbic SP. Foundry: early learnings from the implementation of an integrated youth service network. Early Interv Psychiatry. 2022;16:410–8. 10.1111/eip.13181. 34008340 10.1111/eip.13181 PMC9292689

[36] Hamilton CB, Hoens AM, Backman CL, McKinnon AM, McQuitty S, English K, et al. An empirically based conceptual framework for fostering meaningful patient engagement in research. Health Expect. 2018;21:396–406. 10.1111/hex.12635. 28984405 10.1111/hex.12635 PMC5750689

[37] Hawke LD, Relihan J, Miller J, McCann E, Rong J, Darnay K, et al. Engaging youth in research planning, design and execution: practical recommendations for researchers. Health Expect. 2018;21:944–9. 10.1111/hex.12795. 29858526 10.1111/hex.12795 PMC6250868

[38] Vears DF, Gillam L. Inductive content analysis: a guide for beginning qualitative researchers. Focus Health Prof Education: Multi-Professional J. 2022;23:111–27. 10.11157/fohpe.v23i1.544.

[39] Lumivero. NVivo (Version 12). 2017. https://lumivero.com/products/nvivo/.

[40] Mathieu E, Ritchie H, Rodés-Guirao L, Appel C, Giattino C, Hasell J et al. Coronavirus Pandemic (COVID-19). In: Our World in Data [Internet]. 2020 [cited 15 Nov 2023]. Available: https://ourworldindata.org/coronavirus.

[41] Chiodo D, Lu S, Varatharajan T, Costello J, Rush B, Henderson JL. Barriers and facilitators to the implementation of an integrated youth services network in Ontario. Int J Integr Care. 2022;22. 10.5334/ijic.6737. 10.5334/ijic.6737 PMC9756906 36569415

[42] Statistics Canada. COVID-19 epidemiology update: Current situation. 2023 Nov. https://health-infobase.canada.ca/covid-19/.

[43] Velez G, Hahn M, Troyer B. Making meaning of COVID-19: an exploratory analysis of U.S. adolescent experiences of the pandemic. Transl Issues Psychol Sci. 2022;8:269–81. 10.1037/tps0000326.

[44] Bell IH, Nicholas J, Broomhall A, Bailey E, Bendall S, Boland A, et al. The impact of COVID-19 on youth mental health: a mixed methods survey. Psychiatry Res. 2023;321:115082. 10.1016/j.psychres.2023.115082. 36738592 10.1016/j.psychres.2023.115082 PMC9883078

[45] Dhawan S. Online learning: a panacea in the time of COVID-19 crisis. J Educational Technol Syst. 2020;49:5–22. 10.1177/0047239520934018.

[46] Hamid R, Sentryo I, Hasan S. Online learning and its problems in the Covid-19 emergency period. Jurnal Prima Edukasia. 2020;8:86–95. 10.21831/jpe.v8i1.32165.

[47] Nambiar D. The impact of online learning during COVID-19: students’ and teachers’ perspective. Int J Indian Psychol. 2020;8:783–93.

[48] Hlasny V, AlAzzawi S. Last in after COVID-19: employment prospects of youths during a pandemic recovery. Forum Soc Econ. 2022;51:235–44. 10.1080/07360932.2022.2052738.

[49] Piltch-Loeb R, Merdjanoff A, Meltzer G. Anticipated mental health consequences of COVID-19 in a nationally-representative sample: Context, coverage, and economic consequences. Prev Med (Baltim). 2021;145:106441. 10.1016/j.ypmed.2021.106441. 10.1016/j.ypmed.2021.106441 PMC7838571 33515588

[50] Roberts JD, Dickinson KL, Koebele E, Neuberger L, Banacos N, Blanch-Hartigan D, et al. Clinicians, cooks, and cashiers: examining health equity and the COVID-19 risks to essential workers. Toxicol Ind Health. 2020;36:689–702. 10.1177/0748233720970439. 33241763 10.1177/0748233720970439 PMC7691477

[51] Reny TT, Barreto MA. Xenophobia in the time of pandemic: othering, anti-asian attitudes, and COVID-19. Polit Groups Identities. 2022;10:209–32. 10.1080/21565503.2020.1769693.

[52] Le TK, Cha L, Han H-R, Tseng W. Anti-asian xenophobia and Asian American COVID-19 disparities. Am J Public Health. 2020;110:1371–3. 10.2105/AJPH.2020.305846. 32783714 10.2105/AJPH.2020.305846 PMC7427240

[53] Huang J, Liu R. Xenophobia in America in the age of Coronavirus and beyond. J Vasc Interv Radiol. 2020;31:1187–8. 10.1016/j.jvir.2020.04.020. 32522506 10.1016/j.jvir.2020.04.020 PMC7188638

[54] Dror AA, Eisenbach N, Taiber S, Morozov NG, Mizrachi M, Zigron A, et al. Vaccine hesitancy: the next challenge in the fight against COVID-19. Eur J Epidemiol. 2020;35:775–9. 10.1007/s10654-020-00671-y. 32785815 10.1007/s10654-020-00671-y PMC8851308

[55] Hawke L, LD, DM RJ, SP HJ. Reaching youth with reliable information during the COVID-19 pandemic: social media for sure. Forthcoming.

[56] Murphy J, Vallières F, Bentall RP, Shevlin M, McBride O, Hartman TK, et al. Psychological characteristics associated with COVID-19 vaccine hesitancy and resistance in Ireland and the United Kingdom. Nat Commun. 2021;12:29. 10.1038/s41467-020-20226-9. 33397962 10.1038/s41467-020-20226-9 PMC7782692

[57] Sallam M. COVID-19 vaccine hesitancy worldwide: a concise systematic review of vaccine acceptance rates. Vaccines (Basel). 2021;9:160. 10.3390/vaccines9020160. 33669441 10.3390/vaccines9020160 PMC7920465

[58] Troiano G, Nardi A. Vaccine hesitancy in the era of COVID-19. Public Health. 2021;194:245–51. 10.1016/j.puhe.2021.02.025. 33965796 10.1016/j.puhe.2021.02.025 PMC7931735

[59] Roberts A, Rogers J, Mason R, Siriwardena AN, Hogue T, Whitley GA, et al. Alcohol and other substance use during the COVID-19 pandemic: a systematic review. Drug Alcohol Depend. 2021;229:109150. 10.1016/j.drugalcdep.2021.109150. 34749198 10.1016/j.drugalcdep.2021.109150 PMC8559994

[60] Loades ME, Chatburn E, Higson-Sweeney N, Reynolds S, Shafran R, Brigden A, et al. Rapid systematic review: the impact of social isolation and loneliness on the mental health of children and adolescents in the context of COVID-19. J Am Acad Child Adolesc Psychiatry. 2020;59:1218–e12393. 10.1016/j.jaac.2020.05.009. 32504808 10.1016/j.jaac.2020.05.009 PMC7267797

[61] O’Sullivan K, Clark S, McGrane A, Rock N, Burke L, Boyle N, et al. A qualitative study of child and adolescent mental health during the COVID-19 pandemic in Ireland. Int J Environ Res Public Health. 2021;18:1062. 10.3390/ijerph18031062. 33504101 10.3390/ijerph18031062 PMC7908364

[62] Pujolar G, Oliver-Anglès A, Vargas I, Vázquez M-L. Changes in access to health services during the COVID-19 pandemic: a scoping review. Int J Environ Res Public Health. 2022;19:1749. 10.3390/ijerph19031749. 35162772 10.3390/ijerph19031749 PMC8834942

[63] Chadi N, Ryan NC, Geoffroy M-C. COVID-19 and the impacts on youth mental health: emerging evidence from longitudinal studies. Can J Public Health. 2022;113:44–52. 10.17269/s41997-021-00567-8. 35089589 10.17269/s41997-021-00567-8 PMC8796598

[64] Vanaken G-J, Danckaerts M. Impact of green space exposure on children’s and adolescents’ mental health: a systematic review. Int J Environ Res Public Health. 2018;15:2668. 10.3390/ijerph15122668. 30486416 10.3390/ijerph15122668 PMC6313536

[65] Beyer KMM, Kaltenbach A, Szabo A, Bogar S, Nieto FJ, Malecki KM. Exposure to neighborhood green space and mental health: evidence from the survey of the health of Wisconsin. Int J Environ Res Public Health. 2014;11:3453–72. 10.3390/ijerph110303453. 24662966 10.3390/ijerph110303453 PMC3987044

[66] Bustamante G, Guzman V, Kobayashi LC, Finlay J. Mental health and well-being in times of COVID-19: a mixed-methods study of the role of neighborhood parks, outdoor spaces, and nature among US older adults. Health Place. 2022;76:102813. 10.1016/j.healthplace.2022.102813. 35623164 10.1016/j.healthplace.2022.102813 PMC9127349

[67] Vazquez C, Valiente C, García FE, Contreras A, Peinado V, Trucharte A, et al. Post-traumatic trowth and stress-related responses during the COVID-19 pandemic in a national representative sample: the role of positive core beliefs about the world and others. J Happiness Stud. 2021;22:2915–35. 10.1007/s10902-020-00352-3. 33456320 10.1007/s10902-020-00352-3 PMC7798377

[68] Menculini G, Albert U, Bianchini V, Carmassi C, Carrà G, Cirulli F, et al. Did we learn something positive out of the COVID-19 pandemic? Post-traumatic growth and mental health in the general population. Eur Psychiatry. 2021;64:e79. 10.1192/j.eurpsy.2021.2263. 10.1192/j.eurpsy.2021.2263 PMC8888429 35000665

[69] Allemang B, Cullen O, Schraeder K, Pintson K, Dimitropoulos G. Recommendations for youth engagement in Canadian mental health research in the context of COVID-19. J Can Acad Child Adolesc Psychiatry. 2021;30:123–30. 33953764 PMC8056963

[70] Efuribe C, Barre-Hemingway M, Vaghefi E, Suleiman AB. Coping with the COVID-19 crisis: a call for youth engagement and the inclusion of young people in matters that affect their lives. J Adolesc Health. 2020;67:16–7. 10.1016/j.jadohealth.2020.04.009. 32402796 10.1016/j.jadohealth.2020.04.009 PMC7177075

[71] Children and Youth in Challenging Contexts Network. Youth engagement: Empowering youth voices to improve services, programs, and policy. 2013. Avaliable from: https://youthdatingviolence.prevnet.ca/wp-content/uploads/2020/01/ydv-tools-yf-youth-engagement-summary-en.pdf.

[72] Moorhead SA, Hazlett DE, Harrison L, Carroll JK, Irwin A, Hoving C. A new dimension of health care: systematic review of the uses, benefits, and limitations of social media for health communication. J Med Internet Res. 2013;15:e85. 10.2196/jmir.1933. 23615206 10.2196/jmir.1933 PMC3636326

[73] Taggart T, Grewe ME, Conserve DF, Gliwa C, Roman Isler M. Social Media and HIV: a systematic review of uses of social media in HIV communication. J Med Internet Res. 2015;17:e248. 10.2196/jmir.4387. 26525289 10.2196/jmir.4387 PMC4642795

[74] Gulliver A, Griffiths KM, Christensen H. Perceived barriers and facilitators to mental health help-seeking in young people: a systematic review. BMC Psychiatry. 2010;10:113. 10.1186/1471-244X-10-113. 21192795 10.1186/1471-244X-10-113 PMC3022639

[75] Ijadi-Maghsoodi R, Bonnet K, Feller S, Nagaran K, Puffer M, Kataoka S. Voices from minority youth on help-seeking and barriers to mental health services: partnering with school-based health centers. Ethn Dis. 2018;28:437–44. 10.18865/ed.28.S2.437. 30202197 10.18865/ed.28.S2.437 PMC6128338

[76] Council of Canadian Academies. Fault lines: Expert panel on the socioeconomic impacts of science and health misinformation. Ottawa. 2023. https://cca-reports.ca/wp-content/uploads/2023/02/Report-Fault-Lines-digital.pdf

