# Supplementary material for: Supporting young people through the COVID-19 pandemic and beyond: a multi-site qualitative longitudinal study
Source: BMC Health Serv Res. 2024 Oct 22;24:1266. doi: 10.1186/s12913-024-11752-z (PMC11494784; doi:10.1186/s12913-024-11752-z)
Supplement: Supplementary file 1 — Supplementary Material 1. [file 12913_2024_11752_MOESM1_ESM.docx]

**Appendix A.** Semi-structured interview guides

T1 Interview Guide:

1. How would you describe your life before COVID-19 started?
   1. Were you in school? Working? How much/often
   2. How was your social life?
   3. Hobbies?/activities
   4. Day in the life?
2. How would you describe your life now, in COVID-19 times?
3. As a young person, how has COVID-19 affected your everyday life?
   1. Has COVID-19 had any positive impacts on your life? What kinds of impacts?
   2. Has COVID-19 had any negative impacts on your life? What kinds of impacts?
4. What would you be doing different in your life now if it weren’t for COVID-19?
5. Many services for youth have changed in response to the COVID-19 pandemic.
   1. What were you accessing before COVID and what has changed for you?
   2. How could the changes have been better?
   3. Have you used virtual services? Why or why not have you accessed? What kind of services? How have they been working? What kind of remote technology have you used? (phone, text, video).
6. How hopeful are you feeling about Canada’s recovery from COVID-19?
7. What activities are meaningful for you or other youth to engage in to make a difference during the COVID-19 crisis?
8. What kinds of informational resources could you or other youth benefit from during the COVID-19 crisis?
9. How do you see your post-pandemic life?
10. As a young person, you may be of thinking about your future, for example school, career path, etc.
    1. How has your planning process changed due to COVID-19?
    2. How have your plans changed?
11. Imagine your life two years from now. How do you think COVID-19 will have impacted your life in five years?
12. Imagine your life five years from now. How do you think COVID-19 will have impacted your life in ten years?
13. How do you think youth see the pandemic and how seriously they are taking things?
14. What positive contributions are youth making to the pandemic situation?
15. Do you think young people can play a role in planning the response to this pandemic and future pandemics? What kind of role? What would this look like?
16. What role do you think youth are playing in decision-making (policy making, service planning) and how has that impacted the pandemic?
17. If you could make any suggestion to improve the pandemic response for youth, what would that be?
    1. Did the government response help you directly? Was the information easy to understand?

T2 Interview Guide:

1. How would you describe your life since the last time you were interviewed? Has anything changed for you?
   1. During the last interview, you were asked about school/college, or if you are employed… how has that changed for you, if at all?
   2. During the last interview, you were asked about your current social life… [you were missing friends, you have maintained an average social life, etc]… has that changed? How?
   3. During your last interview, you were asked about your hobbies or activities you enjoy and may not be able to do. Has that changed for you? How?
   4. Are you living in a rural area, town, or city? [Base next part off of their answer] Do you think living *[rural/in a town/city/village]* made your experience of COVID any different?
2. Since your last interview, as your experiences of life during the COVID-19 pandemic changed in any other way?
   1. Has COVID-19 had any positive impacts on your life since the summer? What kinds of impacts?
   2. Has COVID-19 had any negative impacts on your life since the summer? What kinds of impacts?
3. During your last interview, you were asked what you would be doing differently in your life if it weren’t for COVID-19. What (or what else) would you be doing now if it weren’t for COVID-19?
4. During your last interview, you were asked about services you may have used before/during COVID, and what it’s been like to use them.
   1. Have you been using services during COVID-19? Have you used mental health services, specifically?
   2. Since your last interview, have you continued using this service, or others, on a regular basis? Did you start or stop using any other services since your last interview?
   3. Since your last interview, have there been any additional changes to this service? What are your thoughts on those?
   4. *If relevant,* how was finishing up with that service during COVID for you?
   5. *If relevant and using a new service*
      1. Why did you decide to use these services?
      2. What kind of support were you offered? (e.g., phone, video, F2F)
      3. What did you find most helpful about these services?
      4. What did you find least helpful?
      5. What were your expectations of the service? Were they met?
      6. Were you satisfied with the services/would you recommend to a friend?
   6. Is there anything about that service that you didn’t mention the last time that you would like to talk about?
5. Something we are interested in is how mental health services have changed during the pandemic, including having more experience and capacity in providing online services.
   1. Do you think online services like this should continue to be offered after the pandemic?
   2. What would this look like when in-person services resume?
   3. Over the course of the pandemic, has your opinion about online services changed?
6. Do you have any more ideas about how service providers could make it easier for young people to engage with mental health services?
7. During your last interview, you might remember that you were asked about Canada’s recovery from COVID-19. How hopeful do you feel about that now?
8. Compared to how you felt during your last interview, why are you feeling more hopeful/less hopeful now?
9. What would make you feel more hopeful? Less hopeful?
10. During your last interview, there may not have been a vaccine available for COVID-19. Now vaccines are slowly being rolled out across Canada.
    1. How do you feel about the vaccine?
    2. Where have you been getting information about the vaccine?
    3. Have you gotten it, or do you plan to get it? Why/why not?
    4. What has influenced your opinion about the vaccine?
       1. What might make you excited to get the vaccine?
       2. What might make you hesitant about the vaccine?
    5. How is this affected by your [identity factor]
    6. What about people in your family and social circles? What are they saying about the vaccine? How is this influencing your perspectives?
    7. What kinds of informational materials do youth need to make good decisions about the vaccine?
    8. Do you have any other thoughts to share with us about the vaccine?
11. During your last interview, you were asked about your life after COVID-19. How do you see your post-pandemic life now?
12. Since your last interview, has anything about your plans for after COVID-19?
13. Do you see COVID-19 having lasting impacts in our society? If yes, what would this look like? How would these affect your everyday life?
14. You were asked before, but just in case anything has changed… Imagine your life five years from now. How do you think COVID-19 will have impacted your life in five years?
15. Imagine your life ten years from now. How do you think COVID-19 will have impacted your life in ten years?
16. Finally, you might remember during your last interview, you were asked about how you think young people see the pandemic. Since then, do you think anything has changed about how young people see the pandemic and how seriously they're taking things?
    1. What contributions are young people making now? Negative/positive?
    2. Do you think there is anything different about the role young people are playing in decision-making (policy making, service planning)?
    3. Is there anything else about how young people get information about COVID-19 that you think would be helpful to say?
    4. What would you say now about the role young people play in planning a response to a pandemic?
    5. If you could make any suggestion to improve the pandemic response for young people, what would that be?
17. Is there anything else we haven’t covered that you would like to add or think we would be interested?
18. Do you have any questions?

T3 Interview Guide:

1. How would you describe your life since the last time we spoke? Has anything changed for you?
   1. The last time we spoke you told me you were in school/college/working/unemployed…how has that changed for you, if at all?
   2. Last we spoke you told me [you were missing friends/had an average social life, etc]…has that changed? How?
   3. Last time we spoke, you told me you enjoyed/weren’t able to do [hobbies/activities]…has that changed for you? How?
   4. Are you still living in a rural area, town or city? Do you think living *[rural/in a town/city/village]* made your experience of COVID any different?

**NB. If young person disclosed any relevant identity factor, interviewer will explore how this might have affected their experience throughout the interview**

1. Has your experiences of life during the COVID-19 pandemic changed in any other way since the last time we spoke?
   1. Has COVID-19 had any positive impacts on your life since the we last spoke? What kinds of impacts?
   2. Has COVID-19 had any negative impacts on your life since the we last spoke? What kinds of impacts?
   3. We’ve heard from some young people that grieving has been different during the pandemic, for example grieving for the loss of a loved one. Have you been affected by grief during the pandemic? How has this gone? How has it been different due to the pandemic?
   4. Last time we spoke, Covid-19 rates were quite high in [insert country]. How do you think people with Covid-19 are perceived? Do you think where people are from (I.e. city or countryside) influences their opinion Covid-19?
2. How would you describe your mental health since we last spoke?
   1. General probes: (If it improved, what factors led to that improvement? If it declined, what lead to the decline?)
3. How would you describe your substance use since we last spoke?
   1. General probes: (How has it changed? If it increased, what factors led to that improvement? If it declined, what lead to the decline?)
      1. Remember to probe based on the substance (e.g. cocaine use may decrease, but cannabis use may increase, etc.)
4. There has been a lot of discussion about mental health during COVID-19. How do you think this has affected people’s attitudes about mental health?
   1. General probes: (How have they changed? If improved, what factors led to that improvement? If worse, what lead to the decline?)
      1. Probe stigma
5. The last time we spoke, we talked about what you would be doing differently in your life if it weren’t for COVID-19. What (or what else) would you be doing now if it weren’t for COVID-19?
6. The last time we spoke, we talked about how services for young people have changed in response to the COVID-19 pandemic. (At the time, you told me about what it was like to use [previously mentioned service], before/during COVID…)
   1. Have you continued using this service/started using this service again/finished engaging with this service since we last spoke?
   2. Have there been any additional changes to this service since we last spoke? What are your thoughts on those?
   3. Some new services have been created during COVID-19. Have you heard of any new services in your community? What kind of services (e.g., virtual, in person, specific cultural groups, etc.)? How has this affected your access to services?
   4. *If relevant,* how was finishing up with that service during COVID for you?
   5. *If relevant and using a new service*
      1. Why did you decide to use these services?
      2. What kind of support were you offered? (e.g., phone, video, F2F)
      3. What did you find most helpful about these services?
      4. What did you find least helpful?
      5. What were your expectations of the service? Were they met?
      6. Were you satisfied with the services/would you recommend to a friend?
   6. *If relevant and using any MH service:*
      1. How did the restrictions affect your ability to benefit from the services? For example, if you were learning new skills in therapy, were you able to apply them when we were under restrictions? How might your experience with this have been different due to COVID-19?
   7. Is there anything about that service that we didn’t chat about the last time that you would like to tell me about?
7. The last time we spoke, we talked about how many mental health services now have more experience and capacity to provide online services and whether you thought online services should continue to be available after the pandemic. Has your opinion about this changed at all?
8. Do you have any more ideas about how we could make it easier for young people to engage with mental health services?
9. In addition to using services virtually, many young people are using technology more than they were before the pandemic: personally, at work, in services and in other areas of life. How is this impacting you? Do you think this is a positive or a negative change? Why?
10. You might remember that we talked about Ireland/Canada’s recovery from COVID-19 the last time we spoke. How hopeful do you feel about that now?
11. Why are you feeling more hopeful/less hopeful now that you were when we last spoke?
12. What would make you feel more hopeful? Less hopeful?
13. Since the last time we talked, there have been changes in how available COVID-19 vaccines are and in how many people have received them..
    1. Have you gotten it, or do you plan to get it? Why/why not?
    2. How have your thoughts on the vaccine changed? What caused them to change?
    3. What about people in your family and social circles? Have their perspectives on the vaccine changed? How is this influencing your perspectives?
    4. Do you have any other thoughts to share with us about the vaccine?
14. The last time we met, we talked about your life after COVID-19. How do you see your post-pandemic life now?
15. Has anything about your plans for after COVID-19 changed since the last time we spoke?
16. As society opens back up, we’ll all be returning to in-person activities soon. How do you feel about this? Is there anything about this that makes you feel positive or hopeful? Is there anything about this that makes you feel afraid or nervous?
17. Do you see COVID-19 having lasting impacts in our society? If yes, what would this look like? How would these affect your everyday life?
18. I asked you this question before, but I will ask it again… Imagine your life five years from now. How do you think COVID-19 will have impacted your life in five years?
19. Imagine your life ten years from now. How do you think COVID-19 will have impacted your life in ten years?
20. Finally, you might remember the last time we met we talked about how young people see the pandemic. Do you think anything has changed about how young people see the pandemic and how seriously are they taking things?
21. What contributions are young people making now? Negative/positive?
22. Do you think there is anything different about the role young people are playing in decision-making (policy making, service planning)?
23. What would you say now about the role young people play in planning a response to a pandemic?
24. If you could make any suggestion to improve the pandemic response for young people, what would that be?
25. Is there anything else about how young people get information about COVID-19 that you think would be helpful to say?
26. Is there anything else we haven’t covered that you would like to add or think we should know?
27. Do you have any questions?
